# Warm-core eddy intensifies surface mixotrophic bacterivory and fuels mesopelagic heterotrophic grazing

**DOI:** 10.1038/s42003-026-10438-y

**Published:** 2026-06-08

**Authors:** Ying Wang, Xin Liu, Zhonghua Zhao, Dapeng Xu, Bangqin Huang, Ping Sun

**Affiliations:** 1 State Key Laboratory of Marine Environmental Science, College of Ocean and Earth Sciences, Xiamen University, Xiamen, China; 2 National Observation and Research Station for the Taiwan Strait Marine Ecosystem, Xiamen University, Zhangzhou, China; 3 Key Laboratory of the Ministry of Education for Coastal and Wetland Ecosystem, College of the Environment and Ecology, Xiamen University, Xiamen, China; 4 Fujian Provincial Key Laboratory for Coastal Ecology and Environmental Studies, Xiamen University, Xiamen, China; 5 State Key Laboratory of Marine Resource Utilization in South China Sea, School of Marine Sciences, Hainan University, Haikou, China

**Keywords:** Microbial ecology, Microbial ecology, Microbial biooceanography

## Abstract

Anticyclonic eddies (ACEs) restructure resources and drive trophic cascades, yet depth-resolved effects on nanoflagellate bacterivory remain unclear. We quantify bacterivory by phago-mixotrophic nanophytoplankton (PMNP) and heterotrophic nanoflagellate (HNF) using fluorescent-bead incubations in a warm-core ACE in the South China Sea. Despite a 16% decline in nanoeukaryotes, PMNP abundance increases significantly (2.8× surface, 1.4× deep chlorophyll maximum (DCM), shifts toward larger cells, and elevates bacterial turnover and phago-mixotrophic production. HNF community grazing also increases at both euphotic depths despite similar per-cell rates, yielding impacts comparable to PMNP. Structural equation models link PMNP grazing to surface water-mass properties and nutrients and DCM turbidity, whereas HNF grazing tracks water-mass and prey/virus dynamics. In the mesopelagic zone, HNFs dominate; grazing HNF abundance increases 2.7× at 500 m and is associated with particulate beam attenuation coefficient, an in situ particle proxy. These findings reveal a depth-partitioned response coupling mesoscale forcing to microbial carbon processing.

## Abstract (web summary)

Warm-core eddies enhance surface bacterivory by mixotrophic and heterotrophic nanoflagellates and strengthen deep heterotrophic grazing, linking mesoscale forcing to microbial carbon processing from surface to mesopelagic waters.

## Introduction

Marine phytoplankton fix an amount of carbon comparable to that sequestered by the planet’s terrestrial vegetation, underscoring their central role in the global climate system^1,2^. Within this community, nano-sized flagellates (2–20 µm) constitute major contributors to primary production in oligotrophic waters^3,4^. Many of these cells are phago-mixotrophs, capable of both photosynthesis and ingestion of bacterial prey, conferring metabolic flexibility in nutrient-limited environments^5,6^. This dual nutritional strategy enhances nutrient acquisition under dissolved-nutrient limitation, increases trophic-transfer efficiency, and can promote vertical carbon export^7–9^. Heterotrophic nanoflagellates (HNFs) are estimated to consume 26–93% of daily bacterial production^10,11^, while phago-mixotrophic nanophytoplankton (PMNP) may obtain 25–99% of their carbon via phagotrophy, often outcompeting specialist heterotrophs and dominating bacterivory in nutrient-poor systems^12,13^. These findings highlight the need to determine how environmental variability regulates the balance between heterotrophic and mixotrophic nanoflagellates, an essential step toward predicting microbial contributions to oceanic carbon cycling.

Mesoscale eddies, widespread, transient vortices spanning tens to hundreds of kilometers and persisting for days to months, profoundly reorganize the physical and chemical structure of the upper ocean^14,15^. Anticyclonic (warm‑core) eddies depress the nutricline through downwelling, producing warm, nutrient‑depleted surface layers that suppress net primary production and modify ecological processes extending into the mesopelagic^16–18^. By reshaping nutrient availability and light–temperature regimes, eddies can restructure microbial communities and modulate nanoflagellate grazing^19^. Yet, the differential responses of HNFs and PMNP to anticyclonic forcing remain poorly resolved, particularly regarding whether surface-layer perturbations propagate into deeper waters.

Below the euphotic zone, anticyclonic eddies modulate organic-matter export and redistribute microbial resources^20,21^. The mesopelagic zone, which contains nearly 40% of global prokaryotic biomass and serves as a key zone for bacterial carbon remineralization, also sustains substantial protistan predation^22,23^. However, most eddy-focused studies have concentrated on mesozooplankton, fish, or higher-trophic-level grazers^24–26^, leaving the roles of microbial grazers such as HNFs and PMNP in microbial carbon processing within mesopelagic zones largely unexplored.

To address this gap, we conducted depth- and size-resolved fluorescent-bead incubation experiments within a warm-core anticyclonic eddy and at adjacent reference stations in the South China Sea. At four depths (surface, DCM, 200 m, 500 m) we quantified total nanophytoplankton and total HNF abundances, identified actively grazing cells including both phago-mixotrophic nanophytoplankton and active heterotrophic grazers, and calculated cell-specific grazing rates and community-level grazing effect. Environmental context was provided by hydrography, nutrients, chlorophyll *a*, bacteria, and viruses. Piecewise structural equation models were applied to identify key environmental controls on PMNP and HNF grazing, while in situ optical proxies of particle conditions were used to evaluate mesopelagic associations. We hypothesized that (i) nutrient-depleted, warm-core eddy conditions would enhance PMNP bacterivory in the euphotic zone, and (ii) although ACE are generally associated with reduced surface productivity, eddy-driven particle retention or redistribution could create locally enhanced particle-rich conditions at depth and be associated with stronger HNF grazing in the mesopelagic zone. By comparing PMNP and HNF across depths, we show how warm-core eddies restructure microbial trophodynamics and vertical carbon transfer in the South China Sea.

## Results

### Sampling region and hydrographic characteristics

**Fig. 1:**
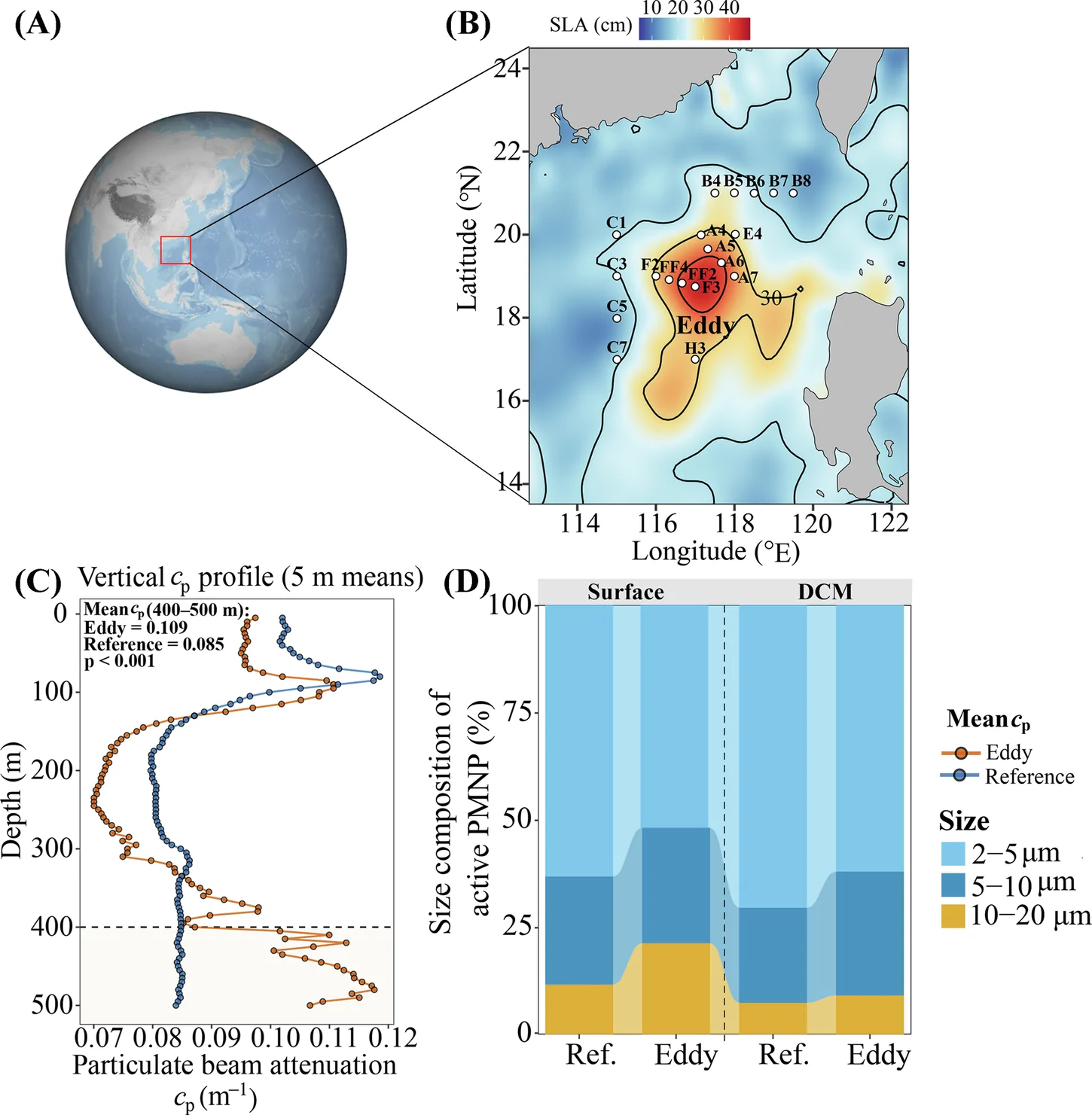
Spatial context, local particle conditions, and phago-mixotrophic nanophytoplankton (PMNP) size structure. **A** Map showing the South China Sea study area (red box). **B** Sea-level anomaly in September 2022 with station locations; the +30 cm contour outlines the warm-core anticyclonic eddy. **C** Vertical profiles of particulate beam attenuation coefficient (*c*_p_) in eddy and reference waters, summarized as 5 m means. *c*_p_ was calculated from beam transmission measured by the CTD-mounted C-Star transmissometer and used as an in situ optical proxy of local particle load. Sample sizes for *c*_p_ profiles are *n* = 2 eddy and *n* = 6 reference stations. **D** Size composition of active phago-mixotrophic nanophytoplankton (PMNP) at the surface (S) and deep chlorophyll maximum (DCM). Sample sizes for size composition analyses are *n* = 9 eddy and *n* = 10 reference per depth.

During the September 2022 cruise, a warm-core anticyclonic eddy (ACE) was occupied the study area (SLA; Figs. 1A, B, S1–2; Supplementary Data 1). We defined the eddy periphery by the +30 cm SLA contour^27^, a threshold supported by tightly packed SLA gradients and in situ anomalies, higher sea-surface temperature and lower salinity, inside the contour (Fig. S1). Along Sections A and F, ACE-driven downwelling depressed the thermocline/halocline from ~60 m near the periphery (A4, F2) to ~80 m toward the core (A6, F3; Section F transected half the eddy). Sections B and C lay outside the eddy and showed only wavy vertical displacements, indicating minimal eddy influence (Fig. S1). Hence, Sections A, F and H3 were treated as eddy stations; Sections B, C, and E4 served as references.

In the euphotic zone, surface temperature was significantly elevated inside the anticyclonic eddy (*p* = 0.033), whereas temperatures at the DCM were slightly higher but not significantly different (*p* > 0.05; Fig. S3). Salinity was significantly lower both at the surface (p = 0.003) and at the DCM (*p* = 0.039), and turbidity decreased significantly at both depths (surface, *p* = 0.029; DCM, *p* = 0.033), indicating enhanced light penetration within the eddy. Nutrient profiles in the upper 200 m were broadly similar between eddy and reference stations, with no significant differences in nutrient concentrations (Fig. S3).

Biological responses within the euphotic zone were depth-dependent but generally modest. Picophytoplankton (*Prochlorococcus*, *Synechococcus*, and picoeukaryotes) tended to be more abundant inside the eddy, although most of these differences were not statistically significant (*p* > 0.05), with the exception of a pronounced surface enrichment of *Prochlorococcus* (*p* = 0.028). Total nanophytoplankton and total heterotrophic nanoflagellate abundances were slightly lower within the eddy at both euphotic depths, but these contrasts were also non-significant (*p* > 0.05; Fig. S3). Surface chlorophyll *a*, bacterial, and viral abundances were modestly reduced in the eddy relative to reference waters, though without statistical support (*p* > 0.05), whereas at the DCM these variables were either slightly elevated (*p* > 0.05) or comparable to the reference stations (Fig. S3).

In the mesopelagic zone (200–500 m), the anticyclonic eddy was associated with more pronounced hydrographic and chemical changes. At 500 m, temperature was significantly higher inside the eddy (*p* = 0.007), whereas turbidity (*p* = 0.032), and nutrient concentrations (all *p* < 0.001) were significantly reduced relative to reference waters (Fig. S4). At 200 m, a similar pattern was observed, although only turbidity differences were statistically significant (*p* = 0.033). For biotic variables, bacterial and viral abundances were slightly lower, while nanophytoplankton and heterotrophic nanoflagellates were slightly higher inside the eddy compared to reference stations, but none of these mesopelagic biomass differences were statistically significant (all *p* > 0.05; Fig. S4). To characterize local particle conditions at depth, we calculated the particulate beam attenuation coefficient (*c*_p_) from beam transmission measured by the CTD-mounted C-Star transmissometer. Vertical *c*_p_ profiles showed that eddy waters tended to become elevated relative to reference waters below approximately 400 m, indicating enhanced deep particle conditions in the eddy interior (Fig. 1C). Collectively, these observations demonstrate that the anticyclonic eddy significantly modified key hydrographic properties (temperature, salinity, turbidity), deep nutrient fields, and local particle conditions, while eliciting more modest, largely non-significant but directionally coherent shifts in microbial standing stocks that varied with depth. This hydrographic-biogeochemical reorganization, together with depth-specific microbial response, provides the physical and biological context for the depth-resolved grazing dynamics described below.

### Nanoflagellate grazing responses

The anticyclonic eddy significantly modulated nanoflagellate grazing in a depth-dependent manner. In the euphotic zone, phago-mixotrophic nanophytoplankton (PMNP) exhibited the most pronounced changes in abundance, proportion of active grazers, and community-level grazing impact, despite an overall 16% decline in total nanoeukaryote abundance (nanophytoplankton + heterotrophic nanoflagellates) inside the eddy (Fig. S5). Below the euphotic zone, bacterivory was sustained almost exclusively by heterotrophic nanoflagellates, which dominated grazing activity in the mesopelagic zone.

### Euphotic zone

**Fig. 2:**
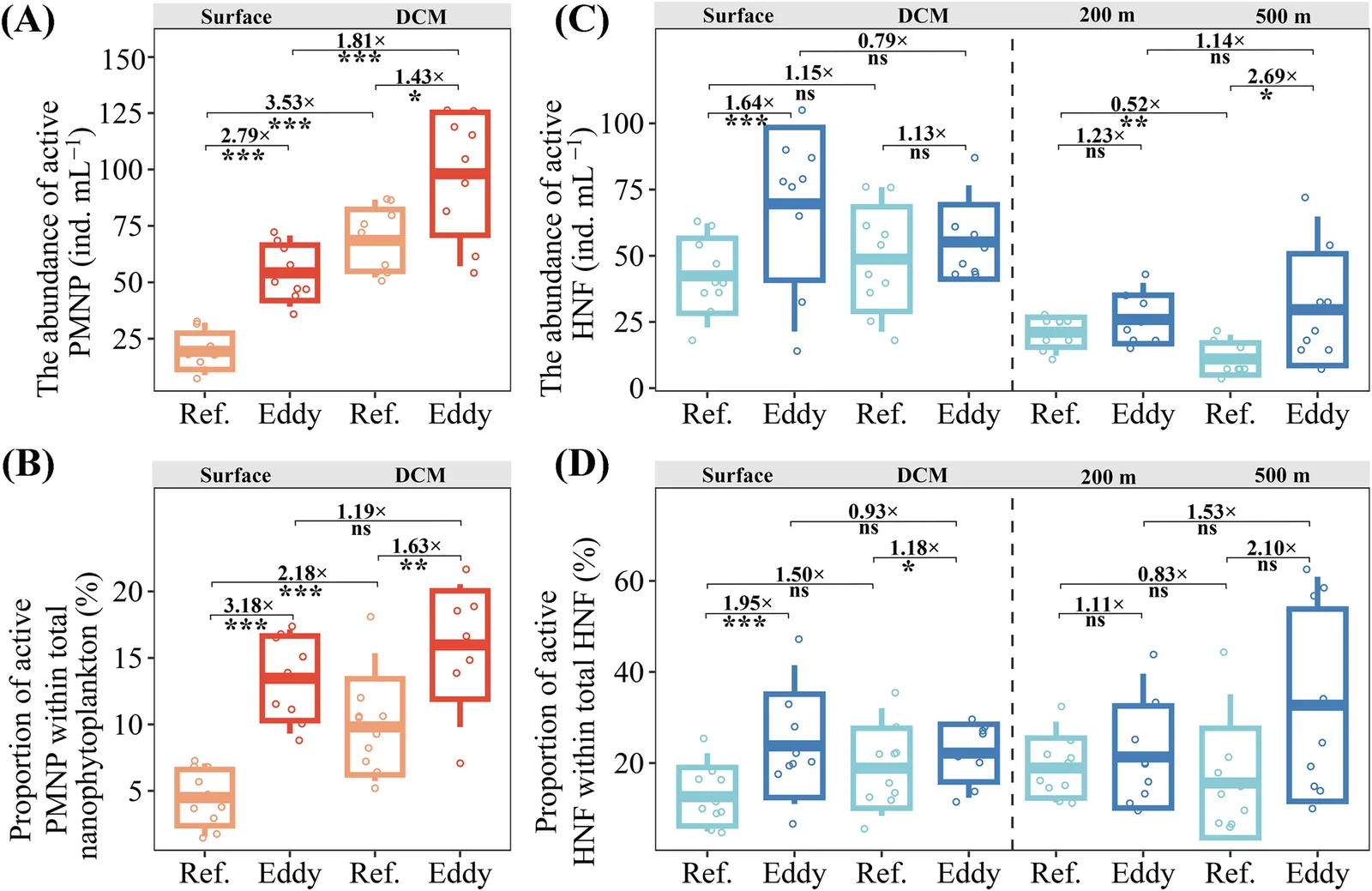
Depth-resolved responses of active phago-mixotrophic nanophytoplankton (PMNP) and heterotrophic nanoflagellate (HNF) to a warm-core anticyclonic eddy. **A**, **C** grazer abundance, **B**, **D** the proportion of active grazers. Each boxplot shows individual station values (*n* = 9 eddy, *n* = 10 reference per depth, except one missing 500 m sample). The central line denotes the mean, boxes represent mean ± standard deviation (SD), and whiskers extend to the 5th and 95th percentiles. Fold changes are shown above brackets, and significance levels indicate pairwise comparisons among depths and between eddy and reference stations within each depth layer (ns, not significant; *p < 0.05; **p < 0.01; ***p < 0.001).

PMNP abundance increased markedly inside the eddy at both euphotic depths (surface: 2.79-fold, *p* < 0.001; DCM: 1.43-fold, *p* = 0.013; Fig. 2A), their relative contribution to the total nanophytoplankton community rose significantly (surface: 4.24% → 13.47%, *p* < 0.001; DCM: 9.82% → 15.98%, *p* = 0.003; Fig. 2B), accompanied by a shift in size structure (Fig. 1D). The relative proportion of 2–5 μm cells declined (surface: 63.26% → 51.87%; DCM: 70.53% → 62.09%), while 5–20 μm cells increased (surface: 11.63% → 21.24%; DCM: 7.37% → 9.07%), driven primarily by 10–20 µm cells at the surface and 5–10 µm cells at the DCM (Fig. 1D; Fig. S6).

**Fig. 3:**
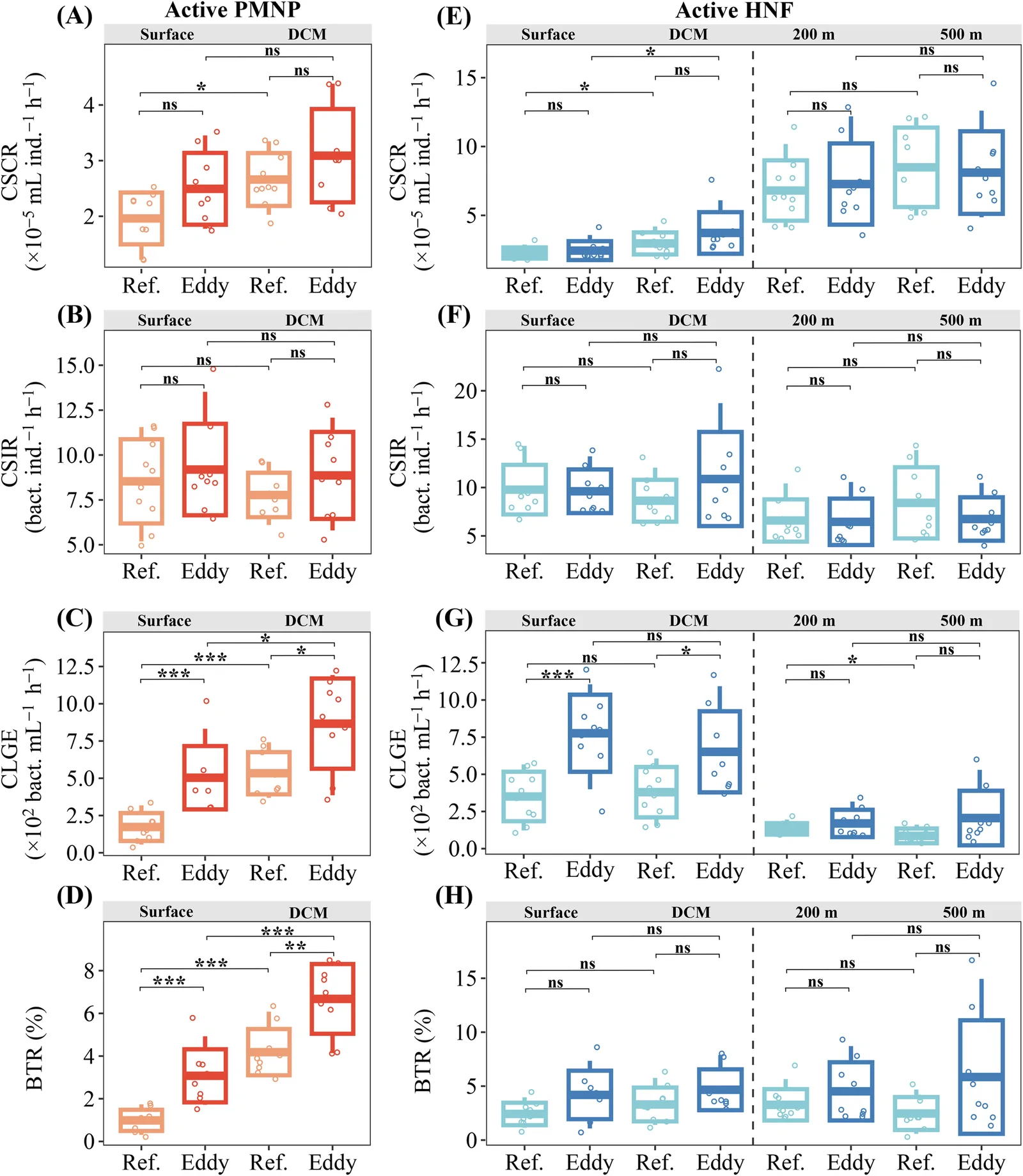
Boxplots of phago-mixotrophic nanophytoplankton (PMNP) and heterotrophic nanoflagellate (HNF) grazing metrics by depth. **A**, **E** cell-specific clearance rate (CSCR), **B**, **F** cell-specific ingestion rate (CSIR), **C**, **G** community-level grazing effect (CLGE), **D**, **H** bacterial turnover rate (BTR). Each boxplot shows individual station values (*n* = 9 eddy, *n* = 10 reference per depth, except one missing 500 m sample). The central line denotes the mean, boxes represent mean ± standard deviation (SD), and whiskers extend to the 5th and 95th percentiles. Ref. reference stations. Significance levels indicate pairwise comparisons among depths and between eddy and reference stations within each depth layer (ns, not significant; *p < 0.05; **p < 0.01; ***p < 0.001).

PMNP did not show a clear difference in physiological grazing activity between eddy and reference waters, as cell-specific clearance rates (CSCR) and ingestion rates (CSIR) were not significantly different (surface CSCR: 19.6 ± 4.6 → 24.9 ± 6.5 × 10^−6^ mL ind.^−^¹ h^−^¹, *p* > 0.05 ; DCM CSCR: 26.6 ± 4.8 → 30.9 ± 8.4 × 10^−6^ mL ind.^−^^1^ h^−^¹, *p* > 0.05; surface CSIR: 8.5 ± 2.3 → 9.2 ± 2.6 bact. ind.⁻^−^; h^−^¹, *p* > 0.05; DCM CSIR: 7.8 ± 1.2 → 8.9 ± 2.4 bact. ind.⁻¹ h⁻¹, *p* > 0.05) (Fig. 3A, B). In contrast, PMNP community-level grazing effects (CLGE) increased significantly (surface: 172.9 ± 93.5 → 503.5 ± 212.6 bact. mL⁻¹ h⁻¹, *p* = 0.001; DCM: 533.8 ± 141.8 → 866.5 ± 302.9 bact. mL⁻¹ h⁻¹, *p* = 0.012; Fig. 3C), reflecting primarily higher PMNP abundance and a shift toward larger size classes rather than enhanced per-cell activity. Consistently, PMNP-mediated bacterial turnover increased from 0.99 to 3.08% d⁻¹ at the surface (3.2-fold, *p* = 0.001) and from 4.18 to 6.68% d⁻¹ at the DCM (1.6-fold, *p* = 0.002; Fig. 3D) indicating that the eddy effect remained significant at the DCM but was smaller in relative magnitude than at the surface.

HNF also responded to eddy conditions. Actively grazing HNF abundance increased 1.64-fold at the surface (42.4 ± 14.2 → 69.6 ± 28.9 ind. mL⁻¹; *p* < 0.001), largely within the small-size classes (Fig. 2C; Fig. S7). At the DCM, however, actively grazing HNF abundance showed no significant difference (48.7 ± 19.8 ind. mL⁻¹→55.2 ± 14.1 ind. mL⁻¹; *p* > 0.05; Fig. 2C). The proportion of actively grazing HNF within the total HNF community nonetheless increased significantly at both depths (surface: 12.19% → 23.80%, *p* = 0.001; DCM: 18.87% → 22.22%, *p* = 0.049; Fig. 2D). HNF CSCR and CSIR remained statistically comparable between eddy and reference stations (surface CSCR: 22.4 ± 4.1 vs. 24.7 ± 6.7 × 10^−6^ mL ind.⁻¹ h⁻¹, *p* > 0.05; DCM CSCR: 28.3 ± 5.9 vs. 38.7 ± 15.3 × 10^−6^ mL ind.⁻¹ h⁻¹, *p* > 0.05; surface CSIR: 9.8 ± 2.6 vs. 9.6 ± 2.3 bact. ind.⁻¹ h⁻¹, *p* > 0.05; DCM CSIR: 8.6 ± 2.2 vs. 10.9 ± 4.8 bact. ind.⁻¹ h⁻¹, *p* > 0.05; Fig. 3E, F). Despite similar per-cell rates, HNF community-level grazing effects increased significantly inside the eddy (surface: 350.8 ± 166.5 → 776.3 ± 259.3 bact. mL⁻¹ h⁻¹, *p* < 0.001; DCM: 379.9 ± 169.4 → 651.4 ± 272.3 bact. mL⁻¹ h⁻¹, *p* = 0.025; Fig. 3G), consistent with elevated abundances and a greater proportion of active grazers. HNF-mediated bacterial turnover showed a modest but non-significant increase from ~2.42–4.18% d⁻¹ to ~3.30–4.66% d⁻¹ across the euphotic zone (*p* > 0.05; Fig. 3H).

Vertical (surface–DCM) comparisons revealed contrasting depth structuring between PMNP and HNF (Figs. 2, 3). For PMNP, grazer abundance, CLGE, and bacterial turnover differed significantly between surface and DCM both inside and outside the eddy (all *p* ≤ 0.01), whereas cell-specific ingestion rates did not. In contrast, HNF exhibited no significant surface–DCM differences in abundance, CLGE, or bacterial turnover (*p* > 0.05), with depth effects restricted to cell-specific clearance rates (*p* < 0.05).

### Mesopelagic zone

Below the euphotic zone, PMNP abundance declined sharply under light limitation, with detectable grazing observed only at 200 m at 10 stations. At this depth, PMNP abundance increased from 1.41 ind. mL⁻¹ at the reference stations to 4.91 ind. mL⁻¹ within the eddy (Fig. S8). However, due to their low abundance and sporadic occurrence, PMNP were excluded from mesopelagic statistical analyses.

HNFs dominated mesopelagic bacterivory. At 200 m, grazing HNF abundance increased modestly inside the eddy (1.23-fold; 21.1 ± 5.6 → 25.9 ± 9.2 ind. mL⁻¹; *p* > 0.05; Fig. 2C), accompanied by a non-significant tendency toward higher bacterial turnover (3.27 ± 1.38% d⁻¹ to 4.66 ± 2.54% d⁻¹, *p* > 0.05; Fig. 3H). Both HNF CSCR and CSIR were comparable between eddy and reference stations (CSCR: 6.8 ± 2.2 × 10^−^⁵ → 7.3 ± 3.0× 10⁻⁵ mL ind.⁻¹ h⁻¹, *p* > 0.05; CSIR: 6.6 ± 2.2 → 6.5 ± 2.4 bact. ind.⁻¹ h⁻¹, *p* > 0.05; Fig. 3E, F). At 500 m, grazing HNF abundance increased 2.69-fold inside the eddy (11.0 ± 6.0 → 29.6 ± 21.1 ind. mL⁻¹; *p* = 0.03; Fig. 2C), representing a statistically significant enhancement in deep grazer abundance. In contrast, HNF CSCR and CSIR showed slight but non-significant decreases (CSCR: 8.5 ± 2.9 × 10⁻⁵ → 8.1 ± 3.0 × 10⁻⁵ mL ind.⁻¹ h⁻¹, *p* > 0.05; CSIR: 8.4 ± 3.7 → 6.8 ± 2.2 bact. ind.⁻¹ h⁻¹, *p* > 0.05; Fig. 3E, F). The higher grazer abundance largely compensated for the modest per-cell declines, resulting in a statistically non-significant increase in community-level grazing effects (CLGE: 86.0 ± 49.0 to 205.9 ± 183.5 bacteria mL⁻¹ h⁻¹, *p* > 0.05; Fig. 3G) and a similar pattern in bacterial turnover (2.46% ± 1.44% to 5.84 ± 4.96% d⁻¹, *p* > 0.05, Fig. 3H).

Vertical (200–500 m) comparisons revealed distinct depth structuring of HNF grazing between reference and eddy stations (Figs. 2C, D and 3E–H). At reference stations, grazing HNF abundance and community-level grazing effect (CLGE) declined significantly from 200 m to 500 m (*p* = 0.002 and *p* = 0.037, respectively), consistent with attenuation of bacterivory with depth, whereas cell-specific grazing rates and bacterial turnover did not differ significantly. In contrast, inside the eddy, no significant 200–500 m differences were detected for any HNF grazing metric (all *p* ≥ 0.18), indicating reduced depth dependence of mesopelagic HNF community-level grazing relative to reference stations.

### Environmental drivers of nanoflagellates grazing

**Fig. 4:**
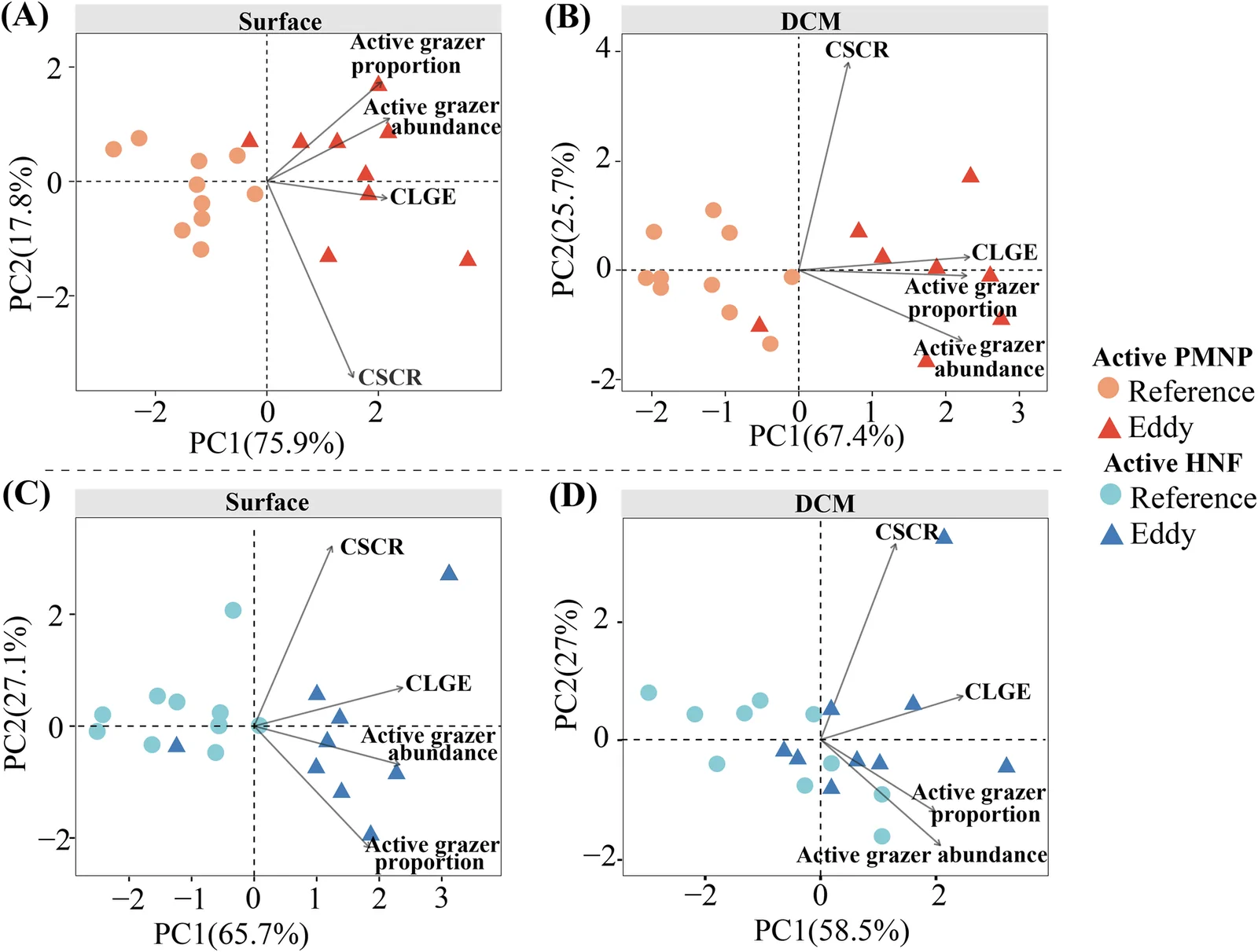
Principal component analysis (PCA) of nanoflagellate grazing activity. PCA biplots show station distributions for (**A**, **B**) phago-mixotrophic nanophytoplankton (PMNP) at the surface and DCM, and **C**, **D** heterotrophic nanoflagellate (HNF) at the surface and DCM. Variables include active grazer abundance, proportion of active grazers, cell-specific clearance rate (CSCR), and community-level grazing effect (CLGE). Each point represents an individual station. Sample sizes: euphotic zone, *n* = 9 eddy and *n* = 10 reference per depth.

**Fig. 5:**
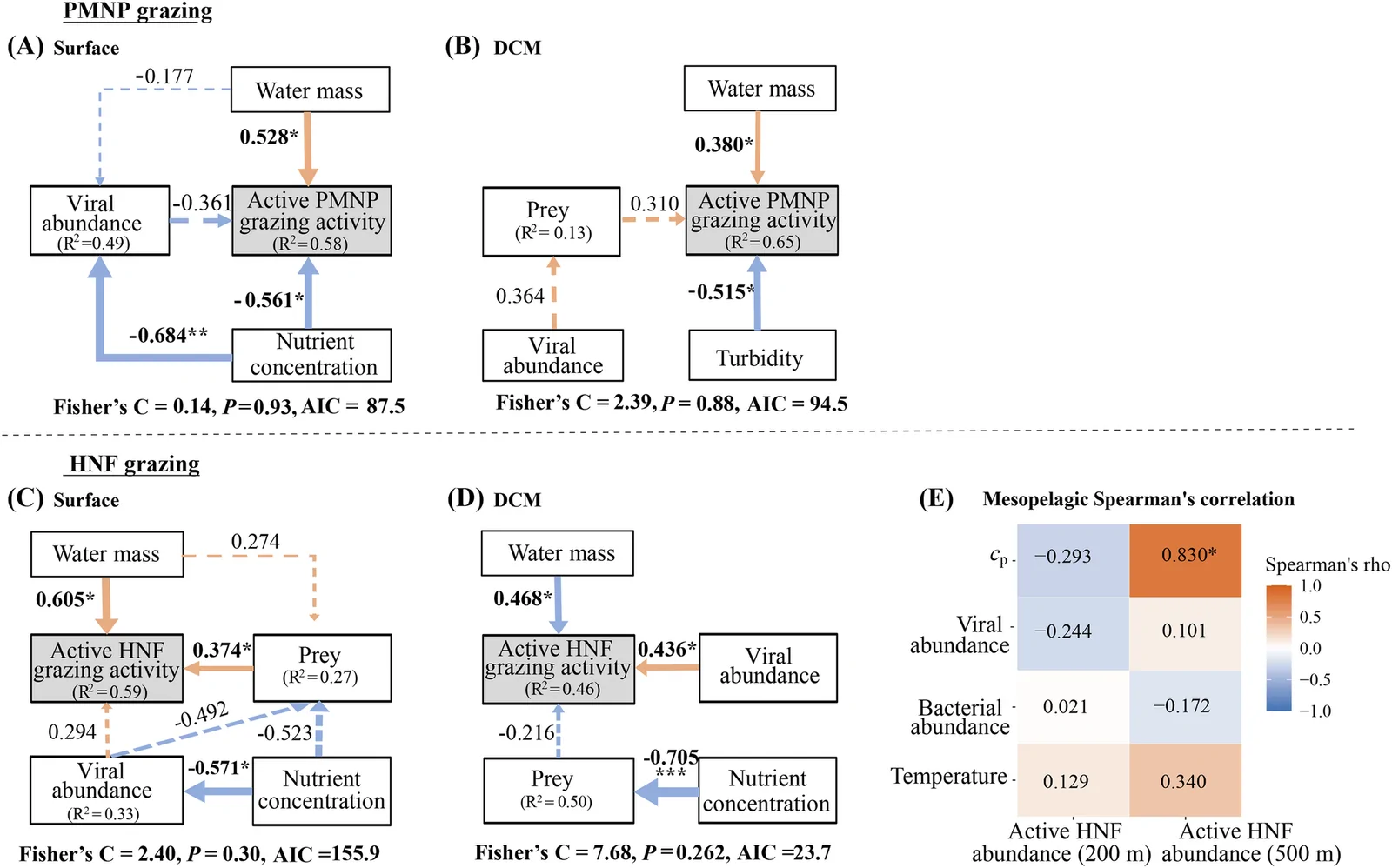
Environmental drivers of phago-mixotrophic nanophytoplankton (PMNP) and heterotrophic nanoflagellate (HNF) grazing across depth layers. **A**, **B** Piecewise structural equation models (piecewiseSEM) showing the main environmental drivers of active PMNP grazing at the surface and deep chlorophyll maximum (DCM). **C**, **D** PiecewiseSEM showing the main environmental drivers of active HNF grazing at the surface and DCM. Sample sizes: euphotic zone, *n* = 9 eddy and *n* = 10 reference per depth. Orange arrows denote positive effects; blue arrows denote negative effects, with width scaled to effect size. Solid lines: *p* < 0.05; dashed: *p* > 0.05. R² indicates variance explained. **E** Spearman correlation heatmap showing relationships between active HNF abundance and environmental variables at 200 and 500 m. Sample sizes: mesopelagic zone, *n* = 2 eddy and *n* = 6 reference per depth. Colors indicate Spearman’s ρ; *p < 0.05. In this study, *c*_p_ was used as an in situ optical proxy of local particle load.

We used piecewise structural equation models (piecewiseSEM) to relate variability in nanoflagellate grazing to environmental gradients in the euphotic zone. Grazing responses were represented by four variables encompassing both cell- and community-level processes: active grazer abundance, proportion of active grazers, cell-specific clearance rate, and community-level grazing effect (Fig. 4). Although absolute differences in temperature, salinity, turbidity and nutrients between eddy and reference waters were modest, these variables nonetheless captured the persistent water-mass structure of the anticyclonic eddy and provided an integrative description of its physical context. Overall, the piecewiseSEM indicated that the anticyclonic eddy modified grazing patterns, but the dominant correlates of grazing varied with depth and grazer type (Fig. 5).

For PMNPs, surface grazing activity (all four grazing metrics) was most strongly and positively associated with water-mass properties (temperature, salinity; standardized path coefficient 0.528, *p* = 0.017), with a weaker, non-significant indirect pathway via viral abundance (0.064, *p* > 0.05). Nutrients exerted a significant negative effect on PMNP grazing (−0.561, *p* = 0.033), while viral abundance also showed a negative but non-significant relationship (−0.361, *p* > 0.05). At the DCM, turbidity emerged as the dominant negative driver of PMNP grazing (−0.515, *p* = 0.006), whereas water-mass properties (0.380, *p* = 0.034) and prey availability (0.310, *p* > 0.05) contributed positively (Fig. 5A, B). For HNFs, surface grazing activity (all four grazing metrics) increased with water-mass properties (0.605, *p* = 0.003) and prey availability (0.374, *p* = 0.047), with viral abundance contributing a weaker, non-significant positive effect (0.294, *p* > 0.05). At the DCM, both viral abundance (0.436, *p* = 0.045) and water-mass properties (0.468, *p* = 0.047) were significant positive predictors of HNF grazing (Fig. 5C, D).

In the mesopelagic zone, we evaluated deep particle associations using *c*_p_ profiles and depth-specific correlation analyses. The strongest particle-related association occurred at 500 m, where active HNF abundance was positively correlated with *c*_p_ (Spearman rho = 0.830, *p* = 0.011; Fig. 5E), whereas no comparable relationship was detected at 200 m (all *p* > 0.05). To assess whether this reflected a coherent deep-layer pattern rather than a single-depth contrast, we also examined the 400–500 m interval using 5 m depth bins. Across this interval, eddy *c*_p_ exceeded reference *c*_p_ in all 21 depth bins, with eddy waters averaging 28.2% higher *c*_p_ than reference waters (Fig. 1C). Together, this depth-specific pattern suggests that local particle conditions were more closely linked to deep grazer abundance at 500 m than at shallower mesopelagic depths.

### Contribution of phago-mixotrophic nanophytoplankton grazing to carbon cycling

**Fig. 6:**
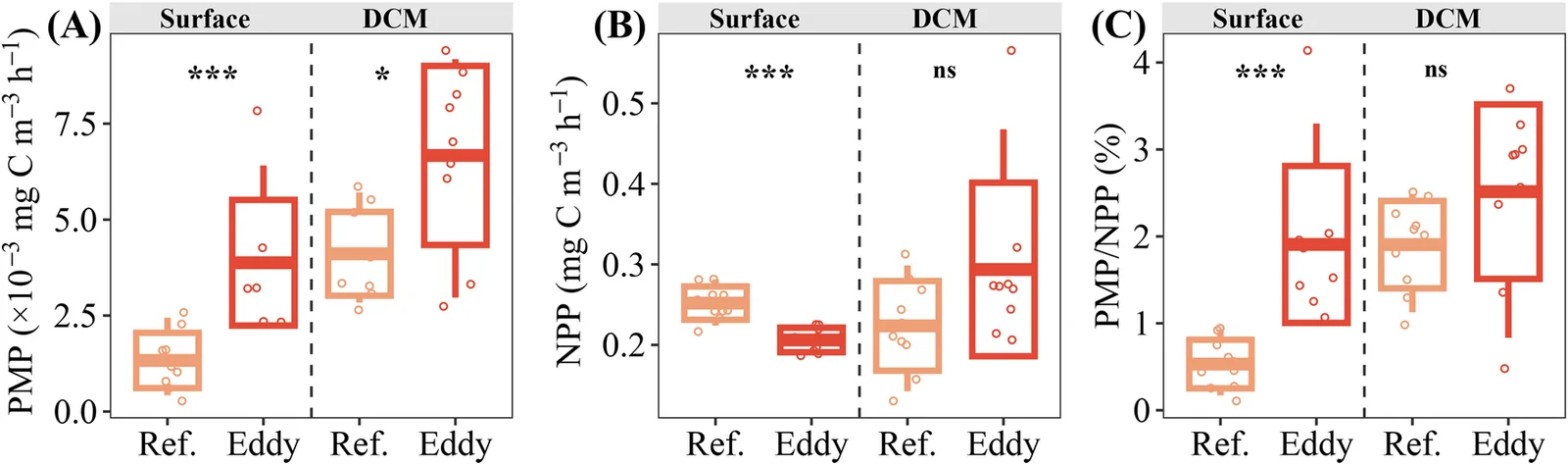
. Boxplots showing (**A**) phago-mixotrophic production (PMP), **B** net primary production (NPP), and **C** PMP:NPP ratio (%) at reference (Ref.) and eddy (Eddy) stations in surface and deep chlorophyll maximum (DCM) waters. Each boxplot shows individual station values (*n* = 9 eddy, *n* = 10 reference per depth). The central line denotes the mean, boxes represent mean ± standard deviation (SD), and whiskers extend to the 5th and 95th percentiles. Significance: ns=not significant; *p < 0.05; ***p < 0.001.

Within the anticyclonic eddy, enhanced PMNP bacterivory increased phago-mixotrophic production (PMP; carbon acquired via ingestion of bacterial prey), thereby strengthening PMNP contributions to microbial carbon cycling. PMP increased nearly threefold at the surface (1.33 ± 0.72 to 3.88 ± 1.64 × 10⁻³ mg C m⁻³ h⁻¹, *p* = 0.001), and 1.6-fold at the DCM (4.11 ± 1.09 to 6.67 ± 2.33 × 10⁻³ mg C m⁻³ h⁻¹, *p* = 0.012; Fig. 6A). In contrast, satellite-derived photoautotrophic net primary production (NPP) showed an opposite and statistically significant surface response, being lower inside the eddy than at reference stations (0.206 ± 0.015 vs. 0.252 ± 0.021 mg C m⁻³ h⁻¹, *p* < 0.001), whereas the DCM increase in NPP inside the eddy was not significant (0.294 ± 0.108 vs. 0.224 ± 0.056 mg C m⁻³ h⁻¹, *p* > 0.05) (Fig. 6B). Consequently, PMP:NPP ratio increased at both depths, more strongly at the surface (≈3.6-fold, 0.53 ± 0.28% to 1.91 ± 0.90%, *p* = 0.001) than at the DCM (≈1.3-fold, 1.91 ± 0.50% to 2.51 ± 1.00%, *p* > 0.05; Fig. 6C).

## Discussion

### PMNP grazing dynamics in the euphotic zone

Consistent with our expectations that oligotrophic, eddy-trapped waters would favor a greater community-level contribution of PMNP, PMNP abundance rose 2.79-fold at the surface (*p* < 0.001), driving a ~ 3.2-fold increase in bacterial turnover (*p* = 0.001). Because PMNP cell-specific clearance and ingestion rates did not differ significantly between eddy and reference waters, we interpret this response primarily as a shift in PMNP abundance, active fraction, and community-level impact rather than as enhanced per-cell phagotrophic activity. The response was strongly size-structured, with large PMNP (10–20 μm) showing the greatest relative increase in abundance (5.12-fold, *p* = 0.023; Fig. S6). This pattern is consistent with previous observations that larger mixotrophs can become more prominent under nutrient-limited conditions^28,29^, although our data do not demonstrate a significant increase in per-cell grazing rates. PiecewiseSEM indicated that surface PMNP grazing correlated most strongly with water-mass properties (temperature and salinity; standardized path = 0.528, *p* = 0.017) and was negatively associated with nutrients (−0.561, *p* = 0.033). The relatively small hydrographic contrasts observed between eddy and reference waters are consistent with the warm-core eddy being in its mature phase during our sampling period (6–24 September 2022)^30^, when mesoscale anomalies are expected to be attenuated relative to the intensification phase but remain expressed as coherent water-mass structure. Accordingly, although the physical and chemical contrasts we measured were modest in absolute magnitude (<0.5 °C temperature difference; salinity differences remained significant at both euphotic depths; nutrient contrasts were largely non-significant), the “water-mass properties” term (temperature–salinity composite) likely captures persistent eddy-associated hydrographic identity rather than transient instantaneous gradients. The significant piecewiseSEM paths therefore reflect consistent covariance between microbial processes and eddy-specific hydrographic conditions within a relatively narrow environmental range, rather than requiring large contemporaneous gradients. More broadly, these biological differences may reflect the persistent ecological development of a relatively coherent eddy water mass, within which temperature and salinity act primarily as hydrographic proxies rather than as strong direct drivers on their own. This interpretation is also compatible with broader observations that mixotroph-dominated assemblages tend to become more prominent in warm and nutrient-poor systems^31,32^, and that larger phytoplankton may become less competitive for scarce nutrients because of declining uptake affinity^6^. In the present study, this pattern is reflected in the increased abundance and community-level impact of PMNP, not in a significant increase in cell-specific grazing rates.

At the DCM, eddy effects on PMNP were also significant but differed in magnitude from those at the surface. PMNP abundance rose 1.43‑fold (*p* = 0.013), community grazing increased ~1.6-fold (*p* = 0.012), and PMNP-mediated bacterial turnover rose from 4.18 to 6.68% d⁻¹ (1.6‑fold, *p* = 0.002; Fig. 3D). Thus, although absolute turnover remained higher at the DCM than at the surface, the relative enhancement of PMNP grazing and turnover inside the eddy was smaller at the DCM than in surface waters. SEM identified turbidity as the dominant negative driver of PMNP grazing at the DCM (standardized path = −0.548, *p* = 0.006), with water-mass properties providing a secondary positive contribution (0.380, *p* = 0.034). Lower turbidity within the eddy likely enhanced light penetration and may have favored the persistence and contribution of PMNP populations at the DCM. This interpretation is broadly consistent with findings from other oligotrophic systems where high light conditions support the prominence of mixotrophs, although our data do not demonstrate a significant increase in per-cell bacterivory^31^.

Eddy-driven increases in PMNP abundance and community-level grazing translated into elevated phago-mixotrophic production (PMP), underscoring their role in channeling bacterial carbon through the microbial loop^33,34^. Ratios of PMP to net primary production (NPP), often used as an indicator of mixotrophic importance^7–9^, were higher inside the eddy (surface ≈ 1.9% vs. 0.5% in reference waters, *p* = 0.001; DCM ≈ 2.5% vs. 1.9%, *p* > 0.05). Here, PMP reflects carbon acquired by phago-mixotrophic nanophytoplankton via ingestion of bacterial prey, whereas NPP represents total phytoplankton production across all size classes. These PMP:NPP ratios are conservative estimates of the relative importance of PMNP bacterivory; using nanophytoplankton-specific NPP would likely yield higher ratios. In addition, the NPP estimates were derived from satellite products with coarser spatial and temporal resolution than our in situ grazing measurements, so small differences should be interpreted cautiously.

Collectively, our results indicate that warm-core eddy conditions favored a larger PMNP assemblage with a greater community-level contribution to bacterivory under relatively warm, low-nutrient, and high-light conditions. The resulting increase in PMNP abundance, active fraction, and size-structured contribution enhanced PMNP-mediated bacterivory and carbon processing at the community level, even though cell-specific grazing rates did not differ significantly. Because the taxonomic composition of the PMNP assemblage was not resolved here, we interpret these changes as shifts in community structure and impact rather than as demonstrated increases in per-cell phagotrophic activity.

### HNF grazing dynamics in the euphotic zone

HNF also responded to the anticyclonic eddy. In the euphotic zone, HNF bacterivory was primarily driven by changes in grazer abundance and in the fraction of active grazers, rather than in per-cell grazing efficiency. Actively grazing HNF abundance increased 1.64‑fold at the surface (*p* < 0.001), accompanied by a significantly higher proportion of active grazers at both euphotic depths (*p* < 0.05), even though total HNF abundance tended to be lower inside the eddy than at reference stations (*p* > 0.05; Figs. 2C, D, S3M). As a result, HNF community-level grazing increased significantly by more than twofold at the surface and ~1.7-fold at the DCM, indicating that enhanced community grazing resulted mainly from a greater number and fraction of actively grazers, rather than from individual-level physiological adjustments.

The piecewiseSEM revealed a clear depth-dependent shift in the dominant microbial and physical correlates of HNF grazing activity. At the surface, HNF grazing covaried positively with prey abundance, as expected for prey-limited consumers, and with water-mass properties (temperature and salinity). At the DCM, however, the direct path from prey abundance to HNF grazing was not significant, whereas viral abundance and water-mass properties emerged as significant positive correlates. We interpret the viral path not as a direct regulatory effect of viruses on HNF grazing, but as an indication of virus–bacteria–grazer coupling: elevated viral abundances generally reflect higher bacterial lysis and turnover, releasing labile organic matter, stimulating bacterial production, and altering prey quality and size structure^35–38^. Additionally, HNFs and ciliates have been shown to graze directly on free viruses or virus-infected bacteria in marine and freshwater systems^39–41^, suggesting that viruses and virus-modified prey can constitute an additional, albeit minor, resource. Under these conditions, bacterial standing stocks at the DCM may already be sufficient to alleviate prey limitation, making viral abundance a better proxy for microbial turnover processes that sustain HNF bacterivory at depth.

Structural models also highlighted the role of water-mass properties, particularly temperature, as part of the physical-biological coupling that shapes HNF grazing. Water-mass showed a significant positive association with HNF grazing in both surface and DCM models, reflecting the slightly warmer and less saline eddy-core water mass. Although these hydrographic contrasts were modest, they were consistent in direction across eddy stations and reflected the coherent identity of the eddy core rather than strong instantaneous forcing. In this sense, the water-mass term may capture the cumulative ecological history of a relatively persistent water mass as much as its immediate physical properties. We therefore interpret temperature and salinity primarily as hydrographic tracers of eddy-associated habitat structure, within which prey availability, microbial turnover, and grazer population dynamics shape the observed HNF response. A positive association between temperature and HNF grazing remains broadly consistent with ecological metabolic theory^42,43^, but in the present system these environmental variables likely function more as proxies of eddy-associated habitat conditions than as strong direct causal drivers on their own.

Overall, the euphotic-zone response of HNFs under eddy influence underscores their persistent and central role in bacterivory, indicating that they remain a major conduit for bacterial carbon even as PMNPs expand their contribution under eddy-modified conditions. Rather than a replacement of heterotrophs by mixotrophs, the eddy fostered the coexistence of two complementary grazing pathways, one dominated by PMNPs and more tightly coupled to light and nutrient constraints, and another dominated by HNFs and more closely linked to prey dynamics and physical-biological structure, together enhancing bacterivorous carbon processing in the upper ocean.

### Mesopelagic grazing dynamics

Linking mesoscale forcing to mesopelagic processing is critical because most carbon exported from the euphotic zone is remineralized at depth^44^. Below the euphotic zone, mixotrophy become negligible, and bacterivory was dominated by HNFs. Inside the eddy, grazing HNF abundance increased modestly at 200 m (1.23-fold, *p* > 0.05) and more strongly at 500 m (2.69-fold, *p* = 0.031), while single-cell clearance and ingestion rates remained comparable (Fig. 2C). These shifts yielded higher community-level grazing and bacterial turnover at both depths though insignificant. Thus, mesopelagic HNF responses were driven primarily by changes in grazer abundance rather than per-cell efficiency.

Although downwelling in warm-core eddies is often expected to reduce local production, *c*_p_ was elevated in deep eddy waters relative to reference waters, indicating enhanced local particle conditions below ~400 m (*p* < 0.001; Fig. 1C). Because *c*_p_ is an in situ optical proxy of total particle load rather than a direct compositional measurement, this pattern is interpreted conservatively. The elevated *c*_p_ below ~400 m indicates that the eddy modified local particle conditions at depth, but it does not by itself identify the origin, composition, or transport pathway of these particles.

The relationship between active mesopelagic HNF abundance and local particle conditions was depth-specific. At 500 m, active HNF abundance was positively associated with *c*_p_ (Spearman rho = 0.830, *p* = 0.011), indicating that local particle enrichment may have contributed to the deeper HNF response. At 200 m, active HNF grazer abundance tended to be higher inside the eddy, although the eddy-reference difference was not statistically significant. Importantly, this pattern occurred near the *c*_p_ minimum and showed no clear association with *c*_p_, indicating that it cannot be explained by local particle enrichment alone. This pattern may instead reflect additional processes not captured by bulk optical particle load, such as the ecological history of the coherent eddy water mass, unresolved prey-related processes, microbial turnover, and grazer population dynamics^45–47^. We therefore interpret particle-rich conditions as one component of the mesopelagic response, particularly at 500 m, rather than as a general explanation for elevated HNF abundance across mesopelagic depths.

### Evaluation of grazing estimation methodology

Grazing rates were estimated using short (1 h) 0.5-µm fluorescent-bead ingestion assays, a standard approach for quantifying nanoflagellate bacterivory in marine and freshwater systems^48–51^. Short incubations provide a snapshot of feeding activity while minimizing prey depletion and incubation artifacts, although they may underestimate rates for slow-feeding or low-activity taxa, particularly in oligotrophic waters. Fluorescent beads also differ from natural bacteria in surface properties and palatability^52^, and low tracer additions (~15–30% of in-situ bacterial abundance) can further limit encounters. These factors likely render our absolute rates conservative; however, because tracer type, concentration, and incubation duration were standardized across all stations and depths, relative eddy- and depth-related differences remain robust. Potential diel variation in ingestion behavior cannot be fully excluded in short-term grazing assays^53,54^. However, both eddy and reference groups included daytime and nighttime incubations, and additional sensitivity analyses indicated that day/night category did not explain the observed eddy–reference grazing differences.

To place our grazing estimates in context, we compared our cell-specific (clearance and ingestion) and community-level (CLGE and bacterial turnover) values with ranges reported across estuarine, coastal, and open-ocean environments (Supplementary Data 2). Reported nanoflagellate bacterivory spans several orders of magnitude, reflecting both ecological variability (e.g., prey availability, trophic state) and methodological diversity (e.g., beads vs. fluorescently labeled bacteria; short vs. long incubations). Within this variability, our euphotic-zone rates are consistent with values reported from open-ocean and low-productivity systems^12,55–57^. Notably, our mesopelagic HNF ingestion rates fall within the limited published range available for comparable depths in North Atlantic Oceans^56^ and the East China Sea^57^. An expanded comparison of grazing parameters with previously reported ranges across marine environments^23,57^, including detailed values by zone and functional group, is provided in Supplementary Text S1. Overall, these comparisons indicate that our estimated magnitudes are broadly consistent with prior observations, while remaining differences likely reflect both environmental setting and methodological choices.

NPP was derived from satellite products, whereas PMP was quantified from in situ measurements. Accordingly, PMP:NPP is interpreted here as an indicative proxy of the relative importance of mixotrophic carbon acquisition rather than a high-precision budget. In the mesopelagic zone, local particle conditions were evaluated using *c*_p_, an in situ optical proxy of particle load. Although *c*_p_ provides direct local information at the depths where grazing was measured, it does not resolve particle composition or flux, and the processes linking eddy dynamics to deep particle-rich conditions therefore remain to be tested directly. Piecewise SEM is particularly useful for disentangling interrelated physical and biological variables in systems with narrow gradients, but its results should be interpreted as standardized associations consistent with the assumed causal structure, not as direct mechanistic evidence.

This study demonstrates that warm-core anticyclonic eddies restructure nanoflagellate bacterivory in a depth-dependent manner (Fig. S9). In the euphotic zone, both PMNP and HNF contributed substantially to enhanced bacterivory at the community level, whereas cell-specific grazing rates showed little change. PMNP exhibited the strongest shifts in abundance, active fraction, and size structure. In the mesopelagic zone, HNF dominated grazing responses, with eddy-associated increases expressed primarily through changes in grazer abundance rather than per-cell rates. Together, these results show that coherent eddy-associated water-mass structure and deep particle-rich conditions can extend mesoscale forcing into the mesopelagic and reshape microbial carbon processing across depth. These findings provide a new framework for understanding how warm-core eddies couple upper-ocean microbial dynamics to deeper carbon processing.

## Materials and methods

### Sampling and environmental measurements

Sampling was conducted aboard R/V Dongfanghong 3 from 6 to 24 September 2022 in the northern South China Sea (115.00°–119.50° E, 16.01°–21.01° N). Nineteen stations were investigated, including nine stations located within a warm-core anticyclonic eddy identified using satellite altimetry-based sea level anomaly (SLA) sourced from the Copernicus Marine Environment Monitoring Services (CMEMS), and ten located at adjacent reference stations outside the eddy (Figs. 1A–B, S1–2; Supplementary Data 1). According to the temporal evolution of the same eddy system (AE-2022) reported by Li et al.^30^, our sampling period coincided with the eddy’s mature phase, during which hydrographic anomalies had weakened relative to peak intensification but the eddy core remained a coherent warm-core water mass. At each station, seawater was collected at the surface, the deep chlorophyll maximum (DCM), 200 m, and 500 m using 12-L Niskin bottles on a Sea-Bird SBE 911 CTD-rosette (*n* = 75; one 500 m sample missing; Supplementary Data 1). Continuous vertical profiles of temperature, salinity, and turbidity were recorded by the CTD sensors and processed using Sea-Bird SBE Data Processing v7.26.7. Beam transmission (%) was recorded by a WET Labs C-Star transmissometer mounted on the CTD package. The particulate beam attenuation coefficient (*c*_p_) was calculated as *c*_p_ = −ln(T/100)/0.25, where T is beam transmission (%) and 0.25 m is the optical path length. In this study, *c*_p_ was used as an in situ optical proxy of local particle load. The transmissometer was not deployed on all CTD casts throughout the cruise that it was temporarily removed during the H and C transects to accommodate methane measurements, reinstalled before the E transect, and subsequently failed at station A6. Consequently, usable *c*_p_ data were available only for a subset of stations. After matching transmissometer records to the biological sampling depths, *c*_p_ data at both 200 m and 500 m were available for eight stations, including two eddy stations and six reference stations. We therefore used all available matched stations for the depth-specific mesopelagic *c*_p_ analyses. Vertical *c*_p_ profiles were summarized as 5 m means for eddy and reference waters (Fig. 1C), and depth-specific associations between active HNF grazer abundance and environmental variables were evaluated at 200 m and 500 m using Spearman correlation analyses.

Chlorophyll *a* (Chl *a*) concentrations were measured following Zhong et al.^58^ Briefly, 1 L of seawater was filtered onto 25-mm Whatman GF/F filters (nominal pore size 0.7 µm) under <15 kPa vacuum. Filters were wrapped in aluminum foil and stored at −80 °C until analysis. In the laboratory, filters were freeze-dried and extracted in 90% acetone for 16–24 h at −20 °C in the dark. Fluorescence (excitation 436 nm) was measured using a Hitachi F-4700 spectrofluorometer (detection limit = 0.01 µg L⁻¹).

For nutrient analyses, 500 mL seawater samples were filtered through 47-mm Whatman GF/F filters (0.7 µm) under gentle vacuum. Filtrates were stored at −20 °C acid-washed polyethylene bottles until analysis. Dissolved inorganic nitrogen (DIN: nitrate, nitrite, ammonium) and dissolved reactive phosphorus (DRP) were quantified using a Technicon AA3 auto-analyzer following standard colorimetric procedures, with detection limits of 0.03 µmol L⁻¹ for both DIN and DRP. All samples were analyzed in duplicate to ensure precision and reproducibility.

For flow cytometric analyses, seawater was prefiltered through 20-µm nylon mesh and subsamples (1.8 mL) were fixed with cold glutaraldehyde (0.5% final), incubated ~15 min in the dark, flash-frozen in liquid nitrogen, and stored at −80 °C until analysis. Duplicate aliquots were prepared for each analysis.

Picophytoplankton (*Prochlorococcus*, *Synechococcus*, and picoeukaryotes) were enumerated following Marie et al.^59^ using a BD FACSAria flow cytometer. Thawed samples were spiked with 1-µm fluorescent beads (~10⁵ mL⁻¹) as an internal standard. Cells were distinguished based on red (FL3) versus side scatter (SSC)^60^, with further separation by orange fluorescence (FL2) (Fig. S10A).

Heterotrophic bacteria were stained with SYBR Green I (final dilution 1:10,000, 10 min in the dark), spiked with 1-µm fluorescent beads, and enumerated using a BD Accuri C6 flow cytometer (488 nm excitation). Bacteria were discriminated by green fluorescence versus side scatter following standard gating (Fig. S10B).

Virus were diluted 2–10× in Tris–EDTA buffer (pH 8), stained with SYBR Green I (1:20,000 final dilution) at 80 °C for 10 min, cooled to room temperature, and enumerated on a Beckman Coulter Epics Altra II (488 nm excitation; 0.1–1 mL h⁻¹; 100 s acquisition) following Marie et al.^59^. Viral particles were gated by green fluorescence versus side scatter (Fig. S10C).

### Satellite data

Daily and monthly gridded SLA (SEALEVEL_GLO_PHY_L4_MY_008_047; 0.125° × 0.125°) and daily net primary production (NPP; GLOBAL_MULTIYEAR_BGC_001_029; 0.25° × 0.25°) were downloaded from CMEMS for 6–24 September 2022. Positive SLA contours delineated the eddy. NPP was extracted from the depth layers nearest the four sampling depths at each station and used as spatially integrated proxies rather than as station-scale flux estimates.

### Enumeration of nanoflagellates

Seawater subsamples (50 mL) were prefiltered through a 20-µm mesh, fixed with ice-cold glutaraldehyde (0.5% v/v final), and gently vacuum-filtered onto 0.2-µm black polycarbonate filters. Filters were stained with DAPI (1 mg mL⁻¹ final; 10 min in the dark), rinsed with sterile seawater, mounted on glass slides, and stored at −20 °C until analysis. Nanoflagellates were enumerated using an Olympus DP71 epifluorescence microscope at 1000× magnification. Under UV excitation (340–390 nm), DAPI-stained nuclei fluoresced blue, while chloroplast autofluorescence appeared red under blue-light excitation (460–495 nm). Pigmented nano-sized cells (2–20 µm) were classified as nanophytoplankton, whereas non-pigmented cells were classified as heterotrophic nanoflagellates (HNF)^61^. For each station-depth sample, at least 50 microscopic fields of view were examined. Each sample was enumerated in duplicate (two parallel filters), and duplicate values were averaged for subsequent analyses. Counting precision was quantified as the relative percent difference (RPD) between duplicate counts: ${\rm{RPD}}( \% )=\frac{|\left(\right.{x}_{1}-{x}_{2}|}{\bar{x}} \times 100$, where ${x}_{1}$ and ${x}_{2}$ are the duplicate counts and $\bar{x}$ is their mean. The mean RPD across all samples was <15%.

### Grazing incubations

At each station and depth (surface, DCM, 200 m, 500 m), seawater was gently prefiltered through 20-µm mesh. Approximately 4 L of seawater were dispensed into two acid-washed 2.4-L polycarbonate bottles per depth. Fluorescent latex microspheres (0.5 µm diameter; Polysciences) were added at ~15% of in situ bacterial abundance. Incubations were conducted for 1 h under simulated in situ conditions. Incubations were initiated during both daytime (06:00–18:00) and nighttime (18:00–06:00) hours over the course of the cruise, and both eddy and reference stations included day and night incubations (Supplementary Data 1). Surface samples were incubated in on-deck flow-through incubators supplied with surface seawater to maintain ambient temperature. DCM samples were incubated under neutral density films corresponding to ~1% surface irradiance. Mesopelagic samples (200 and 500 m) were incubated in the dark at in situ temperatures in temperature-controlled incubators set to CTD-measured values.

At the end of incubations, 50-mL subsamples were fixed with ice-cold glutaraldehyde (1% final concentration), incubated for 10 min in the dark, and filtered onto 0.2-µm black polycarbonate membranes. Filters were mounted on slides and stored at −20 °C until microscopy. Slides were examined sequentially at 1000× magnification with Olympus immersion oil (Japan; refractive index *n* = 1.518) under: (i) UV excitation to visualize DAPI-stained nuclei, (ii) blue-light excitation to detect ingested fluorescent beads, and (iii) blue-light excitation to confirm chloroplast autofluorescence. Nanoeukaryotic cells containing one or more beads were scored as active bacterivores. Bead-containing pigment-bearing cells were classified as active phago-mixotrophic nanophytoplankton (PMNP), whereas bead-containing non-pigmented cells were classified as grazing HNF. Cell sizes were assigned to three classes (2–5 µm, 5–10 µm, 10–20 µm) using a calibrated ocular micrometer. Grazing incubations were processed in duplicate for each station-depth sample, and replicate values were averaged for subsequent rate calculations (mean RPD < 15%).

### Grazing metrics

Grazing was quantified for PMNP and HNF using metrics describing cell-and community-level grazing including active grazer abundance (cells mL⁻¹; bead-positive cells), fraction active (active grazers/total community abundance; PMNP/total nanophytoplankton for mixotrophs; grazing-HNF/total HNF for heterotrophs), cell-specific ingestion rate (CSIR; bacteria grazer⁻¹ h⁻¹), cell-specific clearance rate (CSCR; mL grazer⁻¹ h⁻¹), and community-level grazing effect (CLGE; bacteria mL⁻¹ h⁻¹). In addition, bacterial turnover rate (BTR; % d⁻¹) was calculated as the fraction of the bacterial standing stock removed per day by grazing^23^.

1. Cell-specific ingestion rate (CSIR; bacteria grazer⁻¹ h⁻¹). For each grazer group, the mean number of ingested beads per cell was determined microscopically at the start and end of incubation. Bead ingestion rate was calculated as:
  $${{IR}}_{\mathrm{bead}}=\frac{({{FLB}}_{t}-{{FLB}}_{0})}{{M}_{t}\times \Delta t}$$
  where FLB_*t*_ and FLB_*0*_ are ingested-bead concentrations at *t* = 1 h and *t* = 0 (FLBs mL^-1^); ${M}_{t}$ is the active grazer abundance (cells mL^-1^); and Δt is incubation duration (1 h). Bead ingestion was converted to an equivalent bacterial ingestion rate by scaling to the ratio of in situ bacterial abundance to bead abundance:
  $${CSIR}={{IR}}_{{bead}}\times \frac{{BA}}{{FLB}}$$
  where BA is in situ bacterial abundance (bacteria mL⁻¹) and the initial bead concentration (FLBs mL^-1^).
2. Cell-specific clearance rate (CSCR, mL grazer^−1^ h^−1^). CSCR represents the volume of water cleared of bacteria per grazer per unit time and was calculated as:
  $${CSCR}=\frac{\mathrm{CSIR}}{{BA}}$$
3. Community-level grazing effect (CLGE, bacteria mL^-1^ h^-1^). CLGE represents the community impact and was calculated by scaling per-cell ingestion to the abundance of actively grazing cells:
  $${\mathrm{CLGE}}={CSIR}\times {M}_{t}$$
  where M_t_ is the grazer abundance (ind. mL^-1^).
4. Bacterial turnover rates (BTR, % d^-1^):
  $${\mathrm{BTR}}=\frac{{CLGE}* 24\,h}{{BA}}* 100 \%$$
5. Phago-mixotrophic production (PMP, mg C m⁻^3^ h⁻¹):

$${\mathrm{PMP}}={\mathrm{CLGE}}* 20\,{f\,g}\,C\,{{cell}}^{-1}* 0.385* {10}^{-6}$$

Phago-mixotrophic production (PMP) of the total nanophytoplankton community, expressed as carbon acquired from bacterial prey. Assuming 20 fg C per bacterial cell^62^ and nanoflagellate gross growth efficiency of 0.385^63^.

### Statistics and reproducibility

All statistical analyses were conducted in R v4.1.2 (R Core Team, 2021). Sample sizes corresponded to the number of independent stations sampled at each depth (eddy, *n* = 9; reference, *n* = 10) across four depths, yielding 75 station–depth samples in total (one 500 m sample missing; Supplementary Data 1). Comparisons between eddy and reference stations were conducted separately at each depth using Wilcoxon rank-sum tests. For microscopy-based enumeration and ingestion scoring, duplicate filters were prepared for each station–depth sample; replicate values were averaged, and duplicate precision was assessed using RPD (see above). Where vertical (between-depth) contrasts were described in the Results, corresponding statistical tests and p-values were provided (summarized in Supplementary Data 2). Boxplots display the mean (center line), mean ± standard deviation (SD, box limits), and whiskers extending to the 5th and 95th percentiles; individual points represent all station values. For structural equation modeling and predictor reduction, environmental variables were log-transformed where appropriate and standardized prior to PCA and piecewiseSEM analyses (details in the following SEM Methods).

### Structural equation modeling (SEM)

Four metrics were quantified to capture cell- and community-level processes:(i) active grazer abundance (cells mL⁻¹), (ii) fraction of active bacterivores within the relevant community (PMNP/total nanophytoplankton for mixotrophs; grazing-HNF/total HNF for heterotrophs), (iii) cell-specific clearance rate (CSCR, mL ind.⁻¹ h⁻¹), and (iv) community-level grazing effect (CLGE, bacteria mL⁻¹ h⁻¹). To integrate these response variables, we applied principal component analysis (PCA) and used the first principal component (PC1; >50% variance explained) as a composite grazing-response index. PCAs were conducted separately for PMNP and HNF datasets. For mesopelagic analyses, only active grazer abundance and CLGE showed clear eddy-related variation; therefore, the mesopelagic HNF grazing index was constructed using these two metrics only, while fraction active and CSCR were not included due to limited variation at depth.

Predictors included in situ measurements of temperature, salinity, turbidity, dissolved nutrients (DIN, DRP), chlorophyll *a*, bacterial abundance, picophytoplankton abundance, and viral abundance. To reduce multicollinearity and summarize correlated predictors, we applied PCA (R package vegan) and used PC1 scores as composite predictors. For euphotic-zone models, three composite predictors were constructed: water-mass properties (temperature, salinity; WaterMass_PC1), prey availability (bacterial abundance, picoeukaryotes, *Synechococcus*, and *Prochlorococcus*; Prey_PC1), and nutrients (DIN and DRP; Nutrient_PC1). Turbidity was retained as an independent predictor representing particle/optical conditions.

We used the R package piecewiseSEM (v2.1.2) to evaluate direct and indirect associations between environmental predictors and the composite grazing-response index^64^. A priori dependency structures were specified separately for PMNP and HNF in the euphotic zone (surface and DCM) (Table S1). Models were simplified by removing unsupported paths while preserving the hypothesized causal ordering. Model selection was guided by Akaike Information Criterion (AIC). Overall model fit was assessed using Fisher’s C statistic, with *p* > 0.05 indicating acceptable fit^64^. The retained standardized path coefficients are shown in Fig. 5.

### Reporting summary

Further information on research design is available in the Nature Research Reporting Summary linked to this article.

## Supplementary information

Suppmental Material

Description of Supplementary Data 1 and 2

Supplementary Data 1

Supplementary Data 2

## Data Availability

Copernicus Marine Environment Monitoring Service (CMEMS) products for SLA and NPP can be accessed at (https://data.marine.copernicus.eu/products). Daily and monthly gridded SLA (SEALEVEL_GLO_PHY_L4_MY_008_047; 0.125° × 0.125°) and daily net primary production (NPP; GLOBAL_MULTIYEAR_BGC_001_029; 0.25° × 0.25°) were downloaded from CMEMS for 6–24 September 2022. All data supporting the findings of this study are available in the Zenodo repository at https://doi.org/10.5281/zenodo.1949277665. Any remaining information can be obtained from the corresponding author upon reasonable request.

## Code availability

All custom R scripts used for data processing, statistical analysis, and figure generation are openly available at https://doi.org/10.5281/zenodo.19492776^65^.

## References

[1] Falkowski, P. G. The role of phytoplankton photosynthesis in global biogeochemical cycles. *Photosynth. Res.* **39**, 235–258 (1994). 24311124 10.1007/BF00014586

[2] Ducklow, H., Steinberg, D. & Buesseler, K. Upper ocean carbon export and the biological pump. *Oceanography* **14**, 50–58 (2001).

[3] Duhamel, S., Kim, E., Sprung, B. & Anderson, O. R. Small pigmented eukaryotes play a major role in carbon cycling in the P-depleted western subtropical North Atlantic, which may be supported by mixotrophy. *Limnol. Oceanogr.* **64**, 2424–2440 (2019).

[4] Bolaños, L. M. et al. Small phytoplankton dominate western North Atlantic biomass. *ISME J.* **14**, 1663–1674 (2020). 32231247 10.1038/s41396-020-0636-0 PMC7305139

[5] Jones, R. I. Mixotrophy in planktonic protists: an overview. *Freshw. Biol.* **45**, 219–226 (2000).

[6] Edwards, K. F. Mixotrophy in nanoflagellates across environmental gradients in the ocean. *Proc. Natl. Acad. Sci. USA* **116**, 6211–6220 (2019). 30760589 10.1073/pnas.1814860116 PMC6442547

[7] Mitra, A. et al. The role of mixotrophic protists in the biological carbon pump. *Biogeosciences* **11**, 995–1005 (2014).

[8] Ward, B. A. & Follows, M. J. Marine mixotrophy increases trophic transfer efficiency, mean organism size, and vertical carbon flux. *Proc. Natl. Acad. Sci. USA* **113**, 2958–2963 (2016). 26831076 10.1073/pnas.1517118113 PMC4801304

[9] Stoecker, D. K., Hansen, P. J., Caron, D. A. & Mitra, A. Mixotrophy in the marine plankton. *Annu. Rev. Mar. Sci.* **9**, 311–335 (2017). 10.1146/annurev-marine-010816-060617 27483121

[10] Bong, C. W. & Lee, C. W. The contribution of heterotrophic nanoflagellate grazing towards bacterial mortality in tropical waters: comparing estuaries and coastal ecosystems. *Mar. Freshw. Res.* **62**, 414–420 (2011).

[11] Livanou, E. et al. Pigmented and heterotrophic nanoflagellates: abundance and grazing on prokaryotic picoplankton in the ultra-oligotrophic Eastern Mediterranean Sea. *Deep Sea Res. Part II Top. Stud. Oceanogr.* **164**, 100–111 (2019).

[12] Unrein, F., Massana, R., Alonso-Sáez, L. & Gasol, J. M. Significant year-round effect of small mixotrophic flagellates on bacterioplankton in an oligotrophic coastal system. *Limnol. Oceanogr.* **52**, 456–469 (2007).

[13] Millette, N. C. et al. Mixoplankton and mixotrophy: future research priorities. *J. Plankton Res.* **45**, 576–596 (2023). 37483910 10.1093/plankt/fbad020 PMC10361813

[14] Chelton, D. B., Schlax, M. G. & Samelson, R. M. Global observations of nonlinear mesoscale eddies. *Prog. Oceanogr.* **91**, 167–216 (2011).

[15] Kang, J., Wang, Y., Huang, S., Pei, L. & Luo, Z. Impacts of mesoscale eddies on biogeochemical variables in the northwest Pacific. *J. Mar. Sci. Eng.* **10**, 1451 (2022).

[16] Xiu, P. & Chai, F. Modeled biogeochemical responses to mesoscale eddies in the South China Sea. *J. Geophys. Res. Oceans* **116**, C10006 (2011).

[17] McGillicuddy, D. J. Mechanisms of physical-biological-biogeochemical interaction at the oceanic mesoscale. *Annu. Rev. Mar. Sci.* **8**, 125–159 (2016). 10.1146/annurev-marine-010814-015606 26359818

[18] He, Q. et al. Eddy-induced chlorophyll anomalies in the western South China Sea. *J. Geophys. Res. Oceans* **124**, 9487–9506 (2019).

[19] Chen, P. W.-Y. et al. Distinct water mass between inside and outside eddy drive changes in prokaryotic growth and mortality in the tropical Pacific Ocean. *Front. Mar. Sci.* **11**, 1443533 (2024).

[20] Baltar, F., Arístegui, J., Gasol, J. M., Lekunberri, I. & Herndl, G. J. Mesoscale eddies: hotspots of prokaryotic activity and differential community structure in the ocean. *ISME J.* **4**, 975–988 (2010). 20357833 10.1038/ismej.2010.33

[21] Della Penna, A. & Gaube, P. Mesoscale eddies structure mesopelagic communities. *Front. Mar. Sci.* **7**, 454 (2020).

[22] Arístegui, J. et al. Sub-regional ecosystem variability in the Canary Current upwelling. *Prog. Oceanogr.* **83**, 33–48 (2009).

[23] Pachiadaki, M. G. et al. In situ grazing experiments apply new technology to gain insights into deep-sea microbial food webs. *Deep Sea Res. Part II Top. Stud. Oceanogr.* **129**, 223–231 (2016).

[24] Braun, C. D., Gaube, P., Sinclair-Taylor, T. H., Skomal, G. B. & Thorrold, S. R. Mesoscale eddies release pelagic sharks from thermal constraints to foraging in the ocean twilight zone. *Proc. Natl. Acad. Sci. USA* **116**, 17187–17192 (2019). 31387979 10.1073/pnas.1903067116 PMC6717292

[25] Arostegui, M. C., Gaube, P., Woodworth-Jefcoats, P. A., Kobayashi, D. R. & Braun, C. D. Anticyclonic eddies aggregate pelagic predators in a subtropical gyre. *Nature* **609**, 535–540 (2022). 36071164 10.1038/s41586-022-05162-6

[26] Wang, Y., Zhang, J., Yu, J., Wu, Q. & Sun, D. Anticyclonic mesoscale eddy induced mesopelagic biomass hotspot in the oligotrophic ocean. *J. Mar. Syst.* **237**, 103831 (2023).

[27] McGillicuddy, D. J. et al. Eddy/wind interactions stimulate extraordinary mid-ocean plankton blooms. *Science* **316**, 1021–1026 (2007). 17510363 10.1126/science.1136256

[28] Wehr, J. D. Nutrient and grazer-mediated effects on picoplankton and size structure in phytoplankton communities. *Int. Rev. Gesamten Hydrobiol. Hydrogr.* **76**, 643–656 (1991).

[29] Hillebrand, H. et al. Cell size as driver and sentinel of phytoplankton community structure and functioning. *Funct. Ecol.* **36**, 276–293 (2022).

[30] Li, X. et al. Regulation of mesoscale eddies on oceanic methane production, oxidation, and emissions. *Glob. Biogeochem. Cycles* **39**, e2025GB008500 (2025).

[31] Chan, Y.-F., Chiang, K.-P., Ku, Y. & Gong, G.-C. Abiotic and biotic factors affecting the ingestion rates of mixotrophic nanoflagellates (Haptophyta). *Microb. Ecol.* **77**, 607–615 (2019). 30187089 10.1007/s00248-018-1249-2

[32] González-Olalla, J. M., Medina-Sánchez, J. M. & Carrillo, P. Mixotrophic trade-off under warming and UVR in a marine and a freshwater alga. *J. Phycol.* **55**, 1028–1040 (2019). 31001833 10.1111/jpy.12865

[33] Azam, F. et al. The ecological role of water-column microbes in the sea. *Mar. Ecol. Prog. Ser.* **10**, 257–263 (1983).

[34] Calbet, A. The trophic roles of microzooplankton in marine systems. *ICES J. Mar. Sci.* **65**, 325–331 (2008).

[35] Motegi, C. et al. Viral control of bacterial growth efficiency in marine pelagic environments. *Limnol. Oceanogr.* **54**, 1901–1910 (2009).

[36] Berdjeb, L., Pollet, T., Domaizon, I. & Jacquet, S. Effect of grazers and viruses on bacterial community structure and production in two contrasting trophic lakes. *BMC Microbiol.* **11**, 88 (2011). 21527043 10.1186/1471-2180-11-88 PMC3114703

[37] Zimmerman, A. E. et al. Metabolic and biogeochemical consequences of viral infection in aquatic ecosystems. *Nat. Rev. Microbiol.* **18**, 21–34 (2020). 31690825 10.1038/s41579-019-0270-x

[38] Yang, J. W. et al. Trade-offs between competitive ability and resistance to top-down control in marine microbes. *mSystems* **8**, e01017–e01022 (2023). 36916988 10.1128/msystems.01017-22 PMC10134844

[39] Deng, L. et al. Grazing of heterotrophic flagellates on viruses is driven by feeding behaviour. *Environ. Microbiol. Rep.* **6**, 325–330 (2014). 24992530 10.1111/1758-2229.12119

[40] Kopylov, A. I., Kosolapov, D. B., Zabotkina, E. A. & Kosolapova, N. G. Trophic relationships between planktonic bacteria, heterotrophic nanoflagellates and viruses in a mesoeutrophic reservoir. *Contemp. Probl. Ecol.* **9**, 297–305 (2016).

[41] DeLong, J. P., Etten, J. L. V., Al-Ameeli, Z., Agarkova, I. V. & Dunigan, D. D. The consumption of viruses returns energy to food chains. *Proc. Natl. Acad. Sci. USA* **120**, e2215000120 (2023). 36574690 10.1073/pnas.2215000120 PMC9910503

[42] Choi, J. W. & Peters, F. Effects of temperature on two psychrophilic ecotypes of a heterotrophic nanoflagellate, *Paraphysomonas imperforata*. *Appl. Environ. Microbiol.* **58**, 593–599 (1992). 16348647 10.1128/aem.58.2.593-599.1992 PMC195289

[43] Corradino, G. L. & Schnetzer, A. Impact of temperature and diet on feeding and growth of a marine heterotrophic nanoflagellate. *J. Plankton Res.* **47**, fbaf022 (2025).

[44] Boyd, P. W., Claustre, H., Levy, M., Siegel, D. A. & Weber, T. Multi-faceted particle pumps drive carbon sequestration in the ocean. *Nature* **568**, 327–335 (2019). 30996317 10.1038/s41586-019-1098-2

[45] Freilich, M. A. et al. 3D intrusions transport active surface microbial assemblages to the dark ocean. *Proc. Natl. Acad. Sci. USA* **121**, e2319937121 (2024). 38696469 10.1073/pnas.2319937121 PMC11087786

[46] Wang, Y. et al. Long-lived subsurface anticyclonic eddy enhances microbial diversity and vertical niche partitioning. *Limnol. Oceanogr.* **71**, e70357 (2026).

[47] Jones-Kellett, A. E. & Follows, M. J. A Lagrangian coherent eddy atlas for biogeochemical applications in the North Pacific Subtropical Gyre. *Earth Syst. Sci. Data* **16**, 1475–1501 (2024).

[48] Beisner, B. E., Grossart, H.-P. & Gasol, J. M. A guide to methods for estimating phago-mixotrophy in nanophytoplankton. *J. Plankton Res.* **41**, 77–89 (2019).

[49] Li, Q., Dong, K., Wang, Y. & Edwards, K. F. Relative importance of bacterivorous mixotrophs in an estuary-coast environment. *Limnol. Oceanogr. Lett.* **9**, 81–91 (2024).

[50] Zhan, Y. et al. Spatiotemporal variations of nanophytoplankton mixotrophic strategies in the estuarine-coastal system of the northern South China Sea. *Mar. Pollut. Bull.* **213**, 117622 (2025). 39914118 10.1016/j.marpolbul.2025.117622

[51] McManus, G. B. & Fuhrman, J. A. Bacterivory in seawater studied with the use of inert fluorescent particles. *Limnol. Oceanogr.* **31**, 420–426 (1986).

[52] Weisse, T. et al. Functional ecology of aquatic phagotrophic protists – Concepts, limitations, and perspectives. *Eur. J. Protistol.* **55**, 50–74 (2016). 27094869 10.1016/j.ejop.2016.03.003

[53] Christaki, U. et al. Dynamic characteristics of *Prochlorococcus* and *Synechococcus* consumption by bacterivorous nanoflagellates. *Microb. Ecol.* **43**, 341–352 (2002). 12037612 10.1007/s00248-002-2002-3

[54] Anderson, R., Jürgens, K. & Hansen, P. J. Mixotrophic phytoflagellate bacterivory field measurements strongly biased by standard approaches: a case study. *Front. Microbiol.* **8**, 1398 (2017). 28798734 10.3389/fmicb.2017.01398 PMC5526857

[55] Tsai, A. et al. Importance of bacterivory by pigmented and heterotrophic nanoflagellates during the warm season in a subtropical western Pacific coastal ecosystem. *Aquat. Microb. Ecol.* **63**, 9–18 (2011).

[56] Rocke, E., Pachiadaki, M. G., Cobban, A., Kujawinski, E. B. & Edgcomb, V. P. Protist community grazing on prokaryotic prey in deep ocean water masses. *PLOS ONE* **10**, e0124505 (2015). 25894547 10.1371/journal.pone.0124505 PMC4404134

[57] Cho, B., Na, S. & Choi, D. Active ingestion of fluorescently labeled bacteria by mesopelagic heterotrophic nanoflagellates in the East Sea, Korea. *Mar. Ecol. Prog. Ser.* **206**, 23–32 (2000).

[58] Zhong, Y. et al. Plankton community responses to pulsed upwelling events in the southern Taiwan Strait. *ICES J. Mar. Sci.* **76**, 2374–2388 (2019).

[59] Marie, D., Simon, N., Guillou, L., Partensky, F. & Vaulot, D. Flow cytometry analysis of marine picoplankton. in *In living color: protocols in flow cytometry and cell sorting* (eds Diamond, R. A. & Demaggio, S.) 421–454 (Springer, 2000).

[60] Jiao, N., Yang, Y., Koshikawa, H. & Watanabe, M. Influence of hydrographic conditions on picoplankton distribution in the East China Sea. *Aquat. Microb. Ecol.* **30**, 37–48 (2002).

[61] Sherr, E. B., Caron, D. A. & Sherr, B. F. Staining of heterotrophic protists for visualization via epifluorescence microscopy. in *Handbook of methods in aquatic microbial ecology* (eds Kemp, P. F., Cole, J. J., Sherr, B. F. & Sherr, E. B.) 213–227 (CRC Press, 1993).

[62] Zang, L. et al. Salinity as a key factor affecting viral activity and life strategies in alpine lakes. *Limnol. Oceanogr.* **69**, 961–975 (2024).

[63] Corradino, G. & Schnetzer, A. Grazing of a heterotrophic nanoflagellate on prokaryote and eukaryote prey: ingestion rates and gross growth efficiency. *Mar. Ecol. Prog. Ser.* **682**, 65–77 (2022).

[64] Lefcheck, J., Byrnes, J. & Grace, J. piecewiseSEM: piecewise structural equation modeling. *R package version* **2**, (2016).

[65] Wang, Y. et al. Grazing data of nanoflagellates—Warm-core eddy intensifies surface mixotrophic bacterivory and fuels mesopelagic heterotrophic grazing [Data set]. Preprint at 10.5281/zenodo.19492776 (2026). PMC13578227 42259896

