# Supplementary material for: Warm-core eddy intensifies surface mixotrophic bacterivory and fuels mesopelagic heterotrophic grazing
Source: Commun Biol. 2026 Jun 8;9:1201. doi: 10.1038/s42003-026-10438-y (PMC13578227; doi:10.1038/s42003-026-10438-y)
Supplement: Supplementary file 1 [file 42003_2026_10438_MOESM1_ESM.pdf]

Warm-core eddy intensifies surface mixotrophic bacterivory and fuels mesopelagic heterotrophic grazing

Ying Wang[1,2,3,4], Xin Liu[1], Zhonghua Zhao[5], Dapeng Xu[1*], Bangqin Huang[1,2*], Ping Sun[2,3,4*]

[1] State Key Laboratory of Marine Environmental Science, College of Ocean and Earth Sciences, Xiamen University, Xiamen 361102, China;
[2] National Observation and Research Station for the Taiwan Strait Marine Ecosystem, Xiamen University, Zhangzhou 363000, China
[3]Key Laboratory of the Ministry of Education for Coastal and Wetland Ecosystem, College of the Environment and Ecology, Xiamen University, Xiamen 361102, China;
[4]Fujian Provincial Key Laboratory for Coastal Ecology and Environmental Studies, Xiamen University, Xiamen 361102, China
[5]State Key Laboratory of Marine Resource Utilization in South China Sea, Hainan University, Haikou 570228, China.

Corresponding authors: Ping Sun, psun@xmu.edu.cn; Bangqin Huang, bqhuang@xmu.edu.cn; Dapeng Xu, dapengxu@xmu.edu.cn

Text S1. Expanded contextual comparison of grazing metrics

To place our measured grazing parameters in context, we summarize reported ranges of cell-specific clearance and ingestion rates, community-level grazing effects, and bacterial turnover across diverse marine environments (Table S3). Reported bacterivory spans several orders of magnitude, reflecting both ecological variability (e.g., prey availability, productivity, particle and nutrient regimes) and methodological differences among studies (e.g., short vs long incubations; use of fluorescent beads vs fluorescently labelled bacteria (FLB); fluorescence in situ hybridization; radioisotope-based proxies; and differing size or taxonomic definitions of "mixotrophs" or "protists"). With these caveats, our estimates generally fall well within published ranges, showing particularly strong consistency for heterotrophic nanoflagellate (HNF) ingestion in the mesopelagic zone, where depth-resolved data remain scarce.

Phago-mixotrophic nanophytoplankton grazing in the euphotic zone

Our phago-mixotrophic nanophytoplankton (PMNP) ingestion rates in the South China Sea eddy system (surface: 8.84 ± 2.40 bacteria grazer^{-1} h^{-1}; DCM: 8.28 ± 1.92 bacteria grazer^{-1} h^{-1}; Table S3) are comparable to those reported for mixotrophic or plastidic protists in other marine environments, including coastal northeast Taiwan[1] and temperate coastal waters[2–4]. They are lower than the highest values observed in estuary-influenced and particle-rich environments such as the Pearl River Estuary (up to 27.2 bacteria grazer^{-1} h^{-1})[5], consistent with elevated prey concentration and encounter rates in productive coastal waters relative to our oligotrophic offshore stations. In a comparable regional study in the northern South China Sea, Xiao et al.[6] reported lower PMNP grazing rates (1.42–2.42 bacteria grazer^{-1} h^{-1}), likely reflecting methodological and taxonomic differences. Their TSA-FISH approach quantified two specific mixotrophic lineages (Haptophyta and Mamiellophyceae), whereas our bead-ingestion assays captured the broader PMNP assemblage.

Community-level grazing effects (CLGE) showed similar patterns to the cell-specific trends. Our PMNP CLGE values (surface: 329 ± 230; DCM: 691 ± 283 bacteria mL^{-1} h^{-1}) are comparable to those reported for mixotrophic assemblages in less productive systems[1,4,7], but lower than values from eutrophic coastal zones or from studies aggregating all protists[8–10]. This

difference is expected because community grazing integrates both grazer abundance and prey availability, which vary strongly among ecosystems, and because the "protist" category in other studies often includes broader trophic guilds and size classes beyond PMNP alone.

Heterotrophic nanoflagellate grazing in the euphotic zone

Our HNF ingestion rates in the euphotic zone (surface: 9.70 ± 2.34; DCM: 9.70 ± 3.76 bacteria grazer^{-1} h^{-1}; Table S3) fall within ranges reported from open-ocean and coastal environments, including DCM values from Atlantic protist communities comprising both heterotrophs and mixotrophs[10], from the Mediterranean and coastal observatories[3,11], and from the East China Sea[12]. Our HNF CLGE (surface: 552 ± 302; DCM: 508 ± 258 bacteria mL^{-1} h^{-1}) and bacterial turnover rates (3–4 % d^{-1}; Table 1) also align with estimates from low- to moderate-productivity regions, remaining below those recorded in eutrophic coastal waters or for whole protist communities[7,9,10].

Mesopelagic HNF grazing in context

Direct measurements of HNF bacterivory below the euphotic zone remain limited, making comparisons particularly valuable. Our mesopelagic HNF ingestion rates (200 m: 6.53 ± 2.22; 500 m: 7.58 ± 3.08 bacteria grazer^{-1} h^{-1}) fall within the ranges reported for the Atlantic[10] and East China Sea[12] at comparable depths (Table S3) and are somewhat higher than estimates from the Mediterranean[11]. These differences likely reflect variation in depth strata, substrate availability, and incubation protocols. As expected, grazing rates generally decrease with depth as prey and particle availability decline, so studies extending into deeper mesopelagic or bathypelagic layers typically report lower ingestion rates than those measured in the upper mesopelagic zone. Methodological differences also contribute that longer or in situ incubations may better preserve ambient pressure and temperature, yielding more representative rates for deep waters.

Despite ecological and methodological variability among published studies, the magnitudes of our clearance and ingestion rates, community-level grazing effects, and bacterial turnover estimates are broadly consistent with existing literature. This concordance supports the interpretation that the eddy-driven patterns we observed, particularly the pronounced

community-level responses linked to changes in active grazer abundance and the fraction of actively grazing cells, reflect genuine ecological dynamics rather than methodological artifacts or rate overestimation. Collectively, these comparisons demonstrate that both mixotrophic and heterotrophic nanoflagellates contribute substantially to bacterial carbon processing under mesoscale disturbance, reinforcing their complementary roles in the microbial food web across the upper and mesopelagic ocean.

Table S1. Conceptual structure and hypothesized pathways of the initial structural equation models (SEM) for euphotic phago-mixotrophic nanophytoplankton (PMNP) and heterotrophic nanoflagellate (HNF). “PC1” terms indicate principal components derived from PCA summarizing related environmental gradients.

Model component	Hypothesized drivers (predictor variables)
Euphotic zone: phago-mixotrophic nanoplankton (PMNP)	
PMNP grazing activity (PC1)	WaterMass_PC1 + Nutrient_PC1 + Turbidity + Virus + Prey_PC1
Prey_PC1	WaterMass_PC1 + Nutrient_PC1 + Turbidity + Virus
Virus	WaterMass_PC1 + Nutrient_PC1
Euphotic zone: heterotrophic nanoflagellates (HNF)	
HNF grazing activity (PC1)	WaterMass_PC1 + Virus + Prey_PC1
Prey_PC1	WaterMass_PC1 + Nutrient_PC1 + Turbidity + Virus
Virus	WaterMass_PC1 + Nutrient_PC1

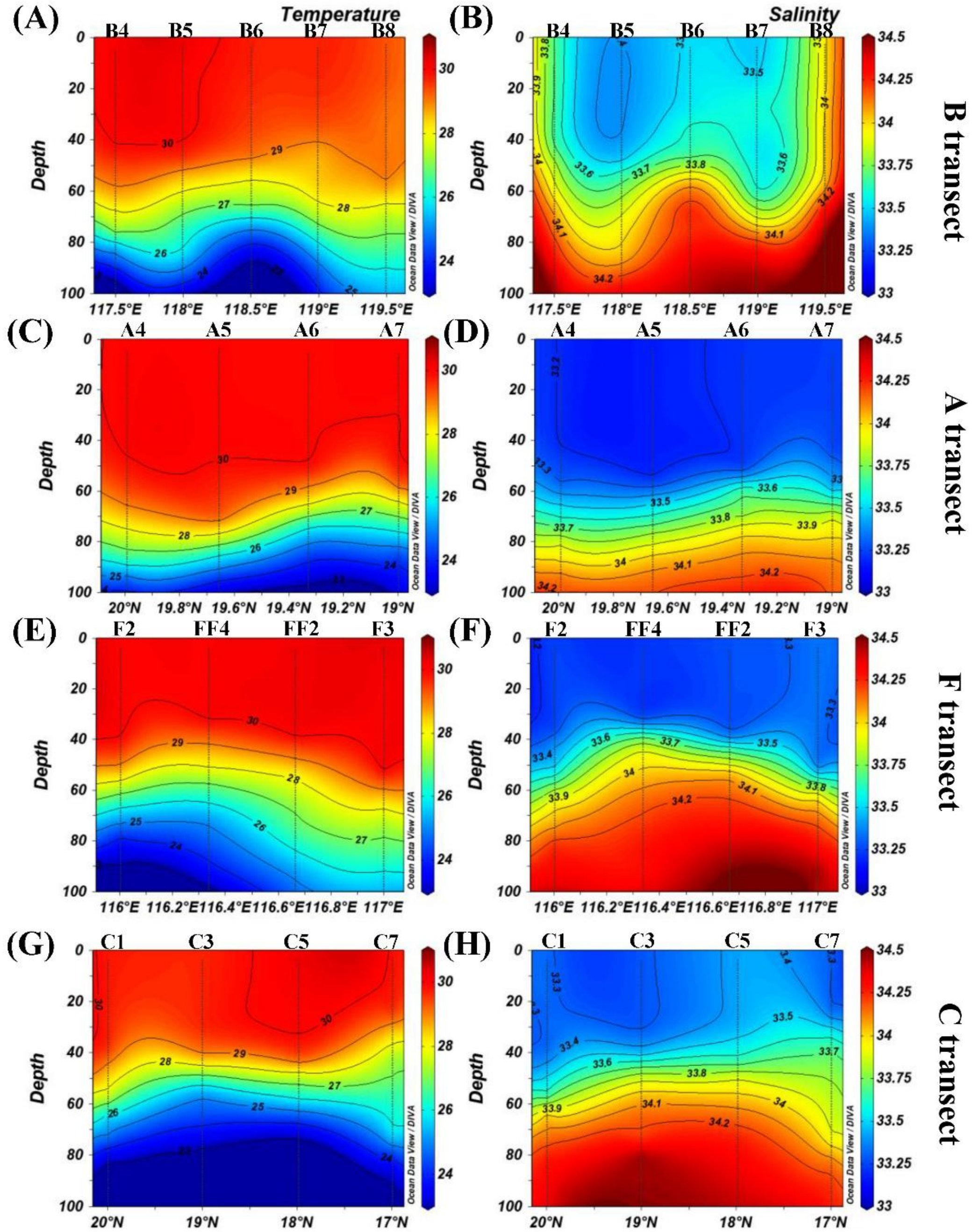

Figure S1. Vertical profiles of temperature and salinity along sampling transects in the South China Sea.

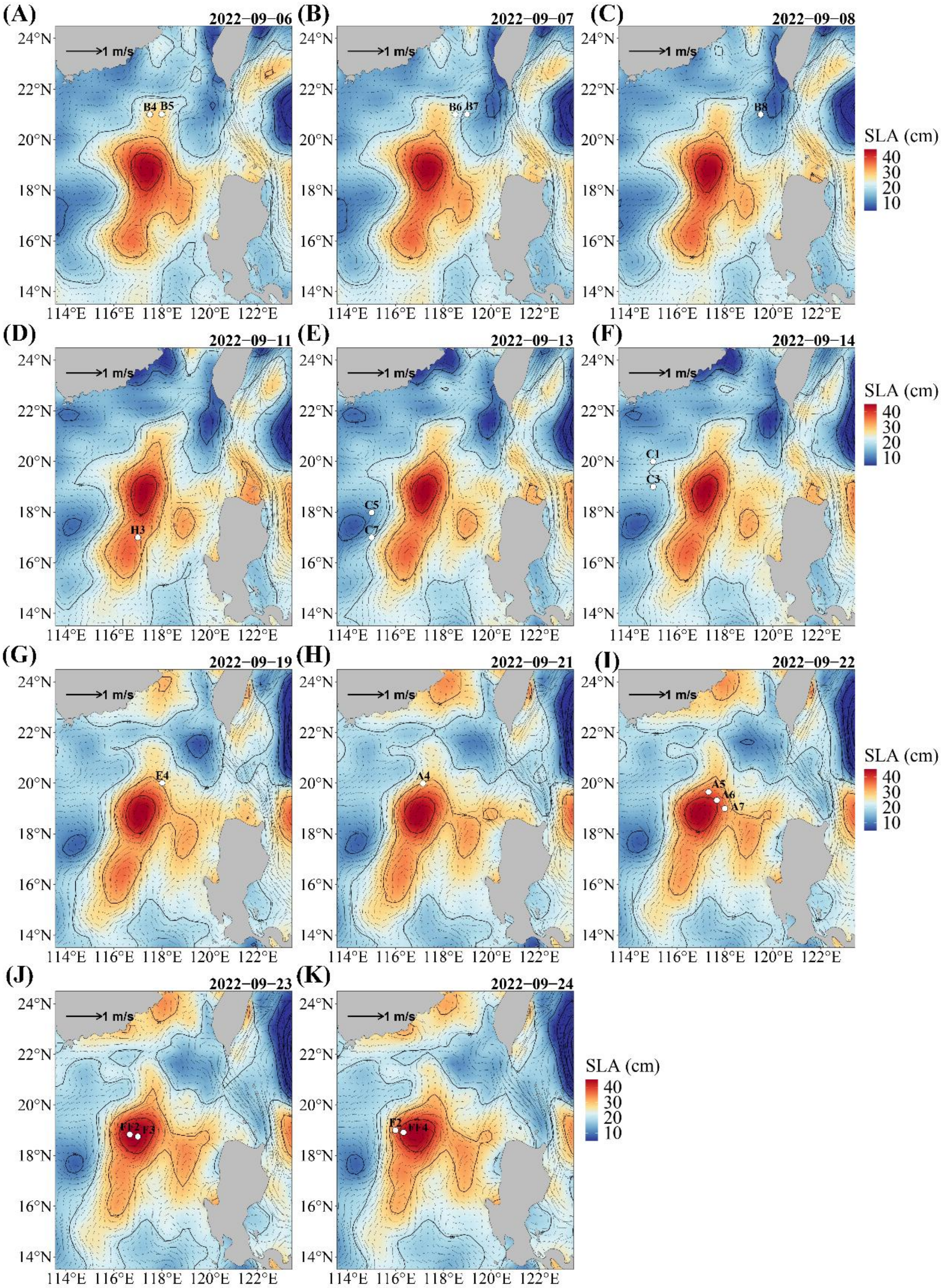

Figure S2. Sea-level anomaly maps for each sampling date with station locations indicated.

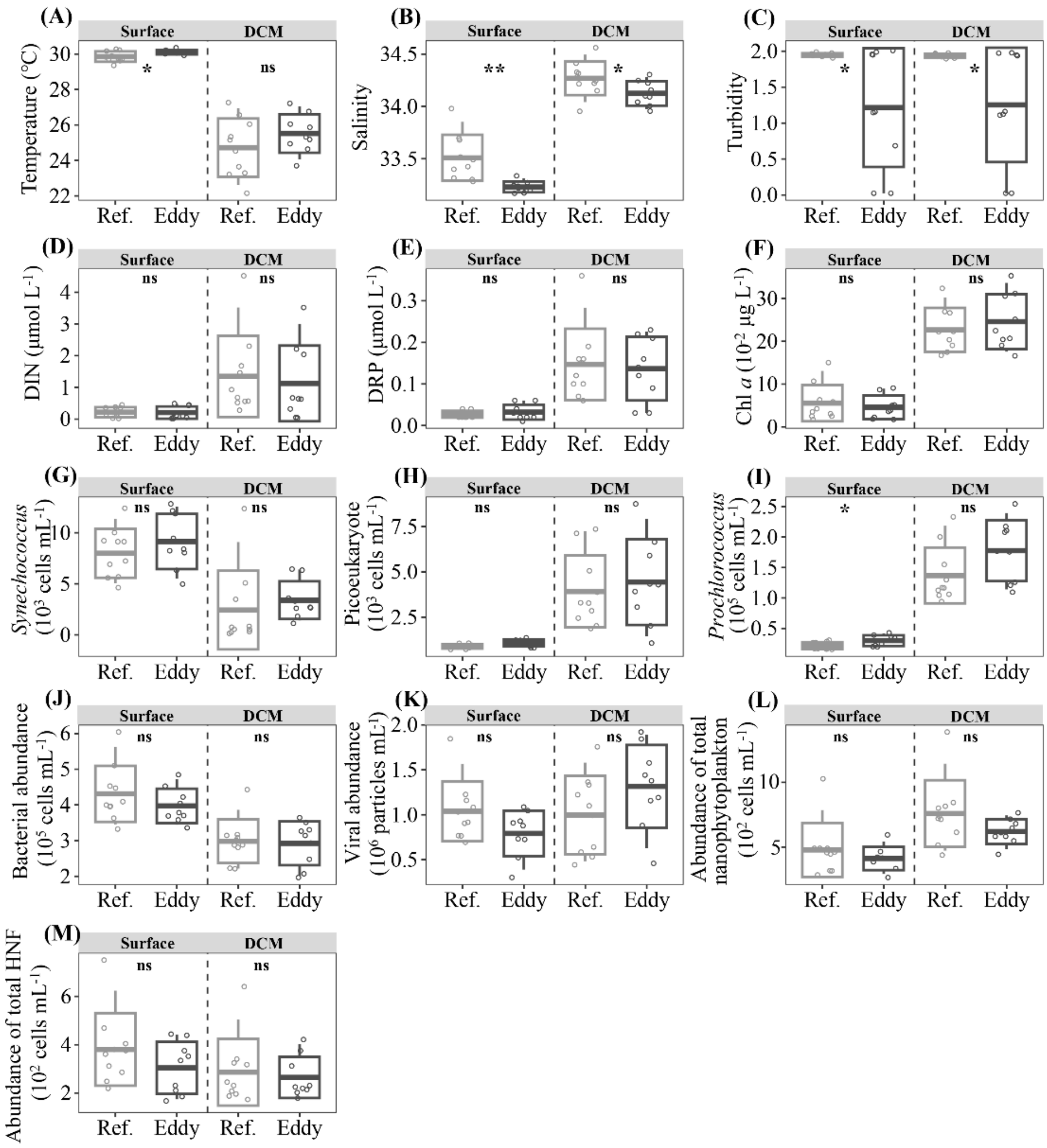

Figure S3. Boxplots of environmental and microbial variables in the euphotic zone (surface and DCM). Reference (Ref.) and eddy (Eddy) stations are compared at each depth. Each boxplot shows individual station values (n=9 eddy, n=10 reference per depth). The central line denotes the mean, boxes represent mean ± standard deviation (SD), and whiskers extend to the 5th and 95th percentiles. Statistical significance is indicated as *** $p < 0.001$; ** $p < 0.01$; * $p < 0.05$; ns, not significant. Abbreviations: Chl *a*, chlorophyll *a*; DCM, deep chlorophyll maximum; DIN, dissolved inorganic nitrogen; DRP, dissolved reactive phosphorus; HNF, total heterotrophic nanoflagellates (all HNF cells counted, irrespective of grazing activity).

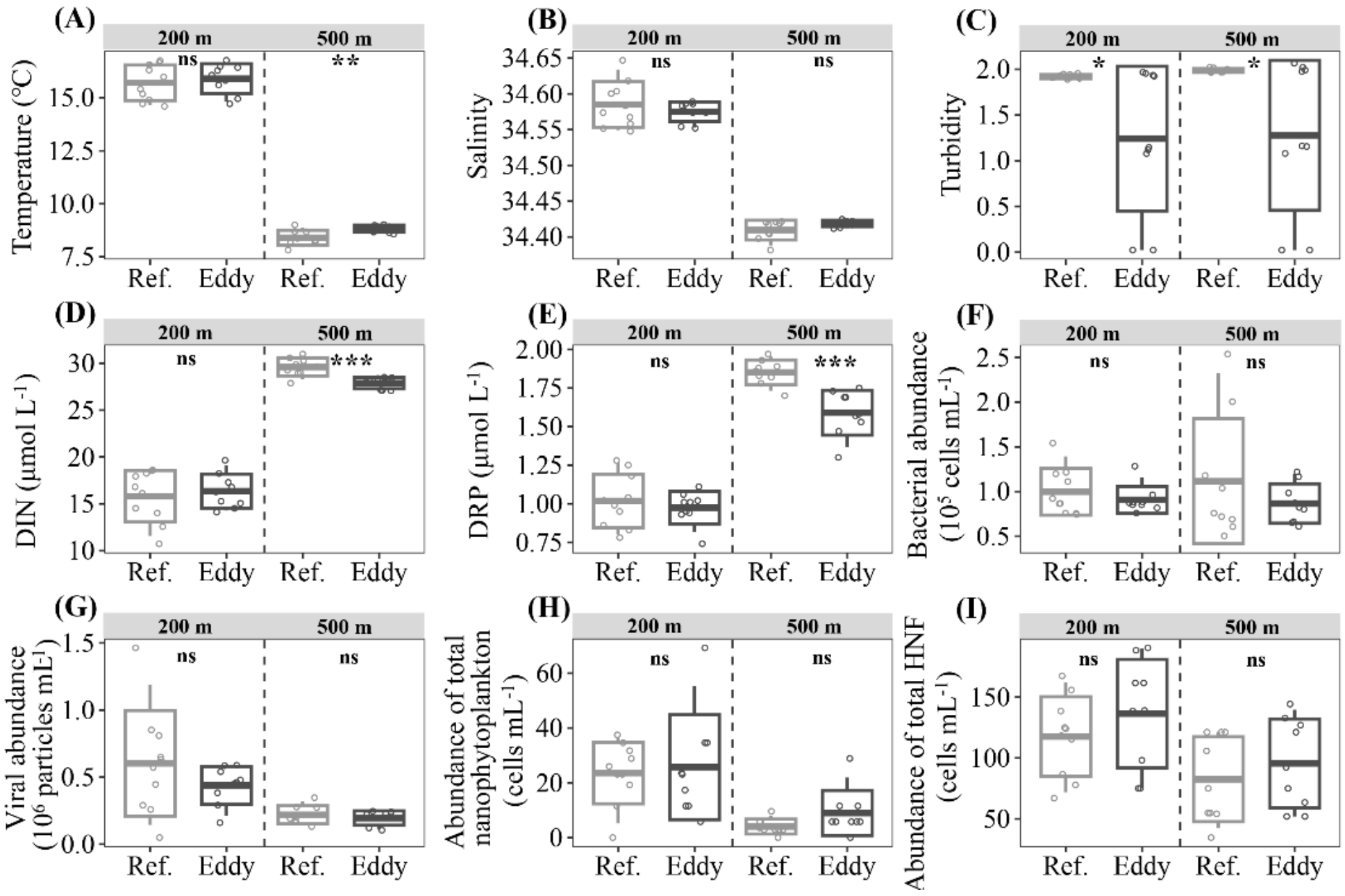

Figure S4. Boxplots of environmental and microbial variables in the mesopelagic zone (200 m and 500 m) at reference (Ref.) and anticyclonic eddy (Eddy) stations. Each boxplot shows individual station values (n=9 eddy, n=10 reference per depth, except one missing 500 m sample). The central line denotes the mean, boxes represent mean ± standard deviation (SD), and whiskers extend to the 5th and 95th percentiles. Statistical significance is indicated as * $p<0.05$; ** $p < 0.01$; *** $p<0.001$; ns, not significant. Abbreviations: DIN, dissolved inorganic nitrogen; DRP, dissolved reactive phosphorus; HNF, total heterotrophic nanoflagellates (all HNF cells counted, irrespective of grazing activity).

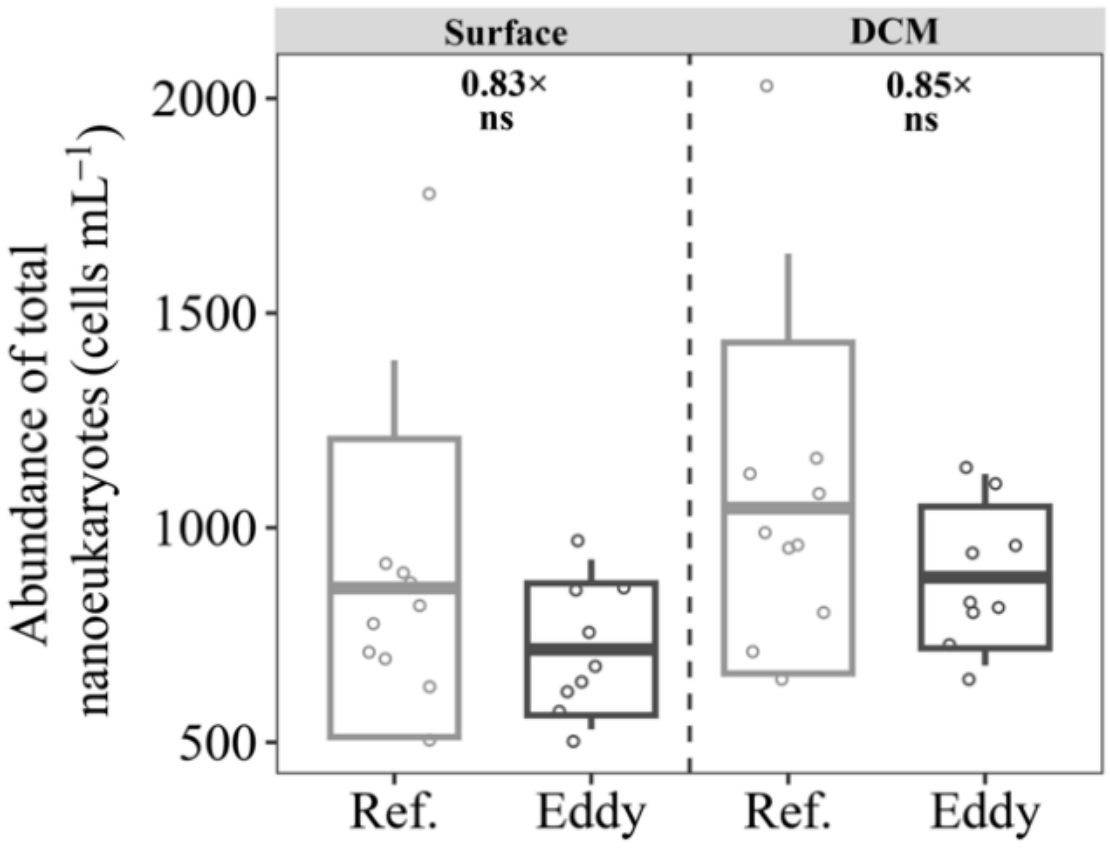

Figure S5. Boxplots of total nanoeukaryotes abundance (nanophytoplankton + heterotrophic flagellates) at reference (Ref.) and eddy (Eddy) stations in the euphotic zone. Numbers above boxes indicate fold change (Eddy/Ref.). Each boxplot shows individual station values (n=9 eddy, n=10 reference per depth). The central line denotes the mean, boxes represent mean ± standard deviation (SD), and whiskers extend to the 5th and 95th percentiles. Statistical significance: ns, not significant. Abbreviation: DCM, deep chlorophyll maximum.

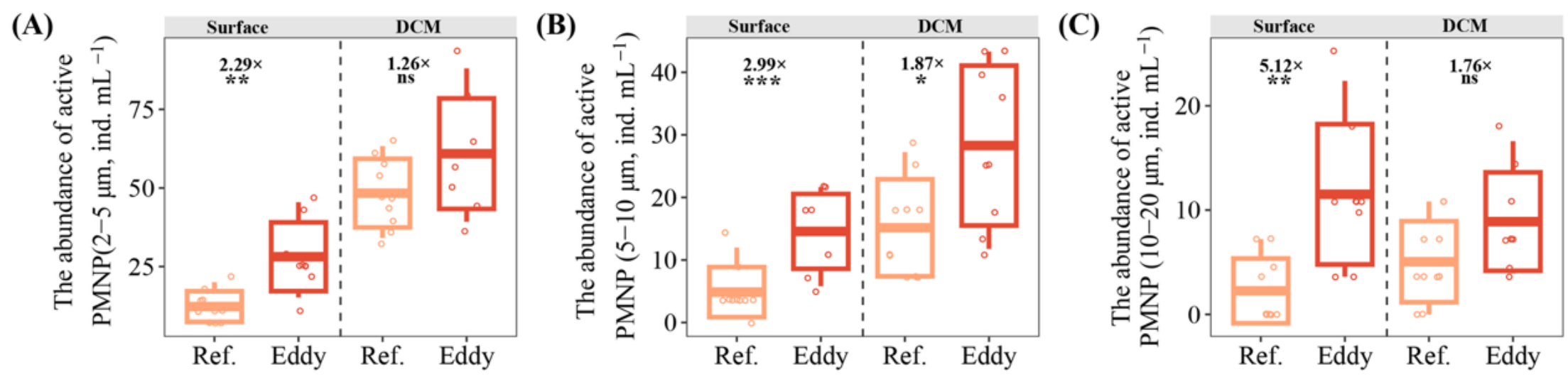

Figure S6. Size-resolved abundances of actively grazing phago-mixotrophic nanophytoplankton (PMNP) at reference (Ref.) and eddy (Eddy) stations in the euphotic zone. Each boxplot shows individual station values (n=9 eddy, n=10 reference per depth). The central line denotes the mean, boxes represent mean ± standard deviation (SD), and whiskers extend to the 5th and 95th percentiles. Size classes are 2–5 µm, 5–10 µm, 10–20 µm. Numbers above boxes indicate fold change (Eddy/Ref.). Statistical significance is indicated as *** $p < 0.001$, * $p < 0.05$; ns, not significant. Abbreviation: DCM, deep chlorophyll maximum.

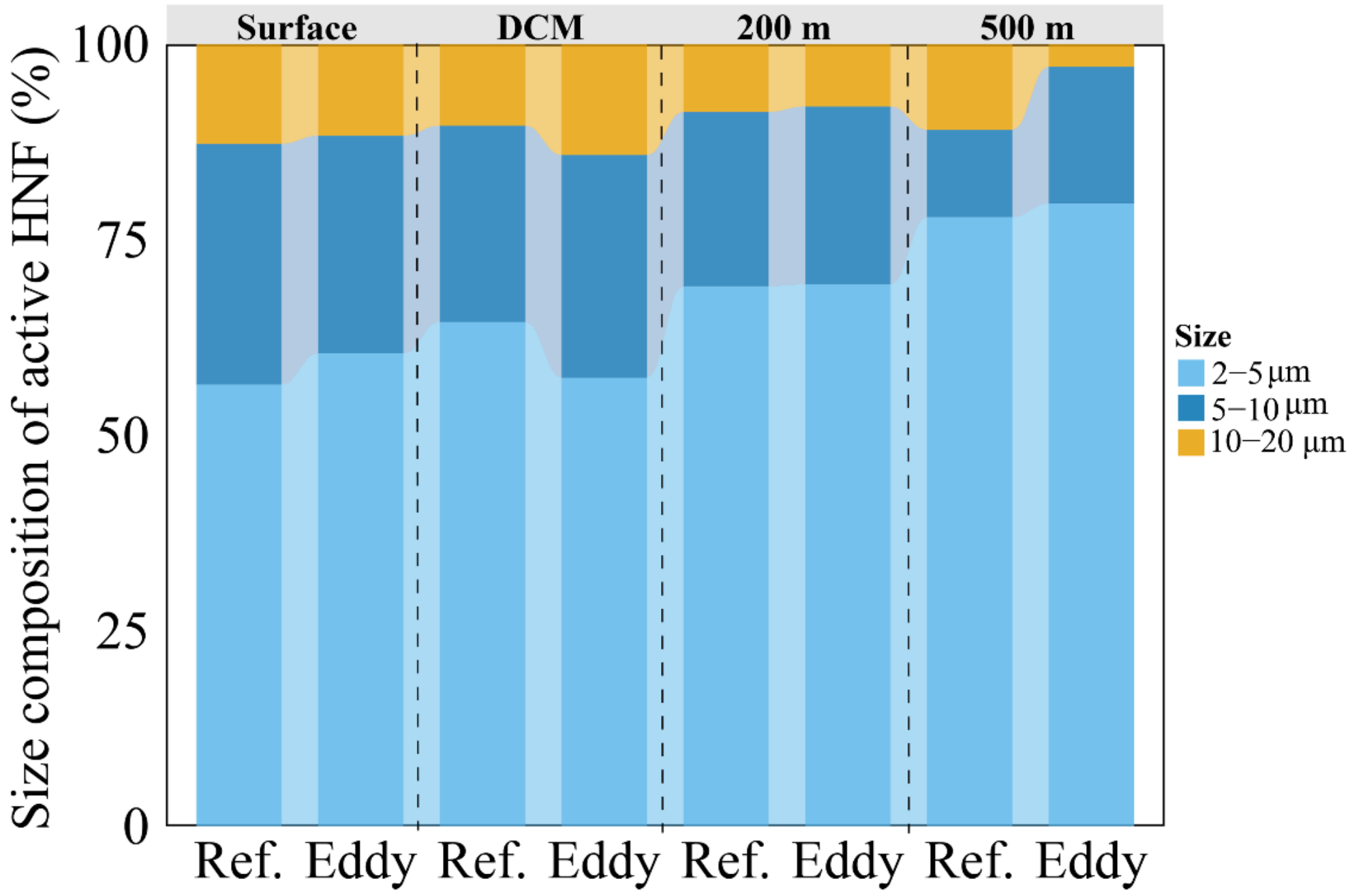

Figure S7. Depth-resolved size composition of actively grazing heterotrophic nanoflagellates (HNF) at reference (Ref.) and eddy (Eddy) stations from the surface to 500 m. Abbreviation: DCM, deep chlorophyll maximum. Sample sizes for size composition analyses are n=9 eddy and n=10 reference per depth, except one missing 500 m sample.

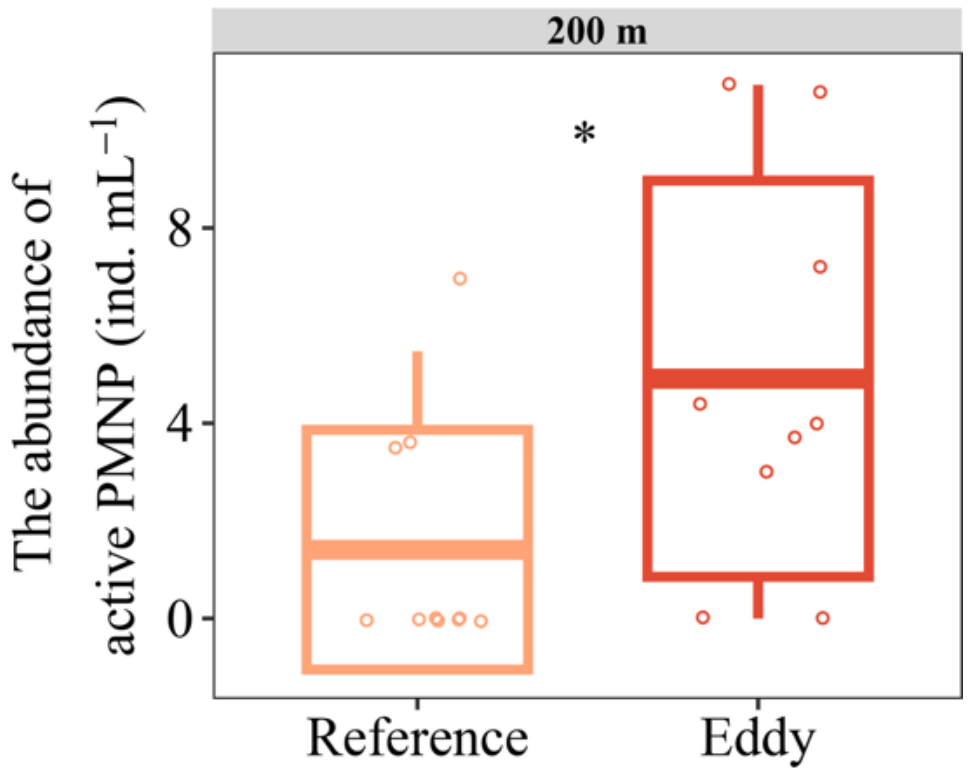

Figure S8. Boxplot of actively grazing phago-mixotrophic nanophytoplankton (PMNP) abundance at 200 m depth at reference and eddy stations. The boxplot shows individual station values (n=9 eddy, n=10 reference at 200 m). The central line denotes the mean, boxes represent mean ± standard deviation (SD), and whiskers extend to the 5th and 95th percentiles. Actively grazing PMNP were detected only sporadically at this depth, and cell numbers were too low for robust mesopelagic statistical analysis. Therefore, these observations were excluded from the mesopelagic PMNP analyses in the main text and are shown here for completeness only. Statistical significance: * $p<0.05$.

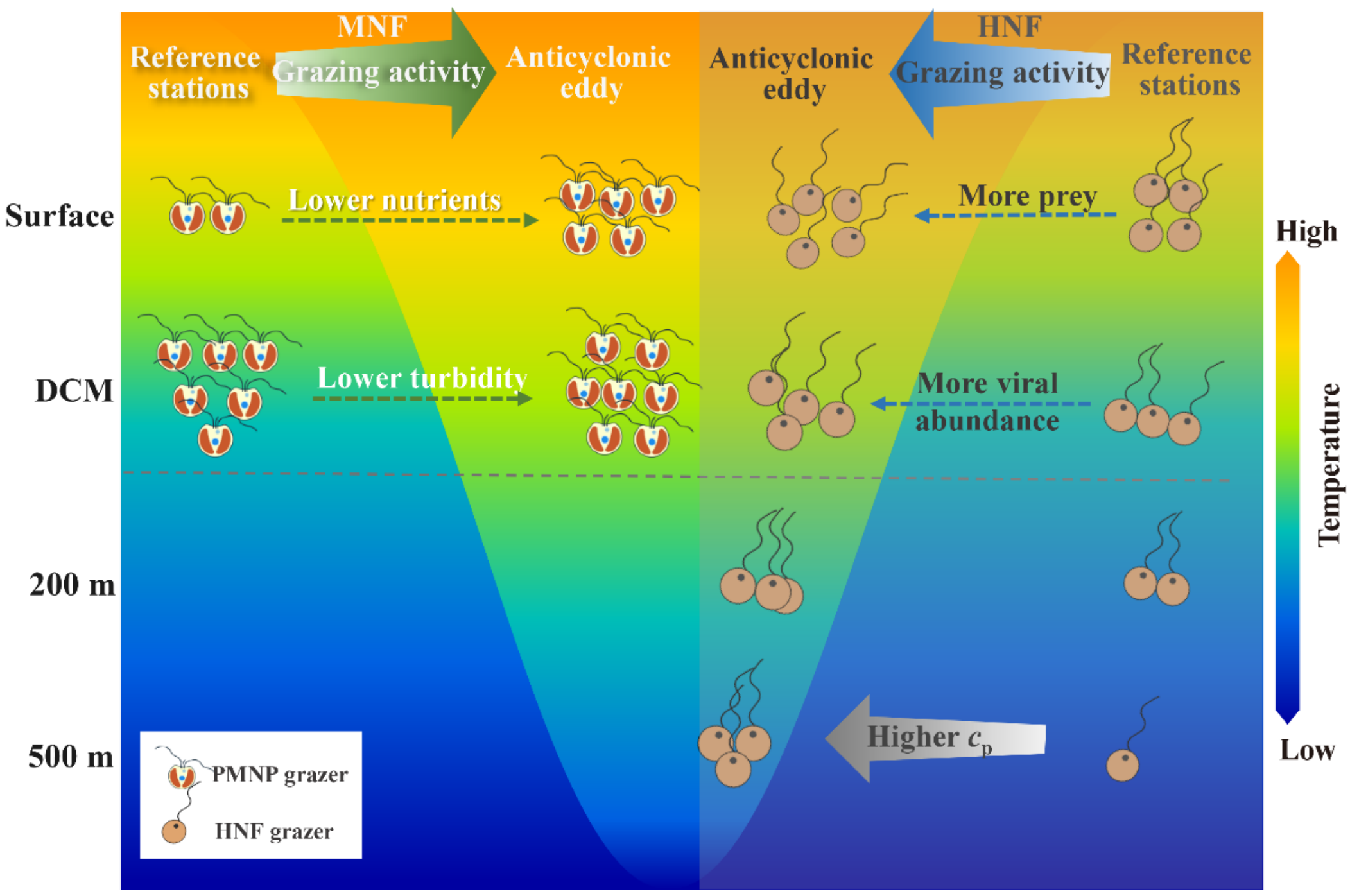

Figure S9. Conceptual synthesis of depth-partitioned grazing dynamics in an anticyclonic eddy. Schematic summary of phago-mixotrophic nanophytoplankton (PMNP) and heterotrophic nanoflagellate (HNF) grazing patterns relative to reference conditions from the surface to 500 m. Background shading represents the vertical temperature structure. In the euphotic zone, the relatively warm, low-nutrient, and low-turbidity water-mass conditions characteristics associated with the eddy core are hypothesized to favor enhanced PMNP grazing and a shift toward larger size classes. At 500 m, active HNF grazer abundance was significantly and positively correlated with particulate beam-transmission-derived c_p, an in situ optical proxy of local particle load. Arrows indicate the dominant environmental associations identified in this study. This schematic represents a conceptual framework and hypothesis, rather than a directly quantified mechanism. Abbreviations: Bac, Bacteria; Pro, *Prochlorococcus*; Syn, *Synechococcus*; Peuk, Picoeukaryotes.

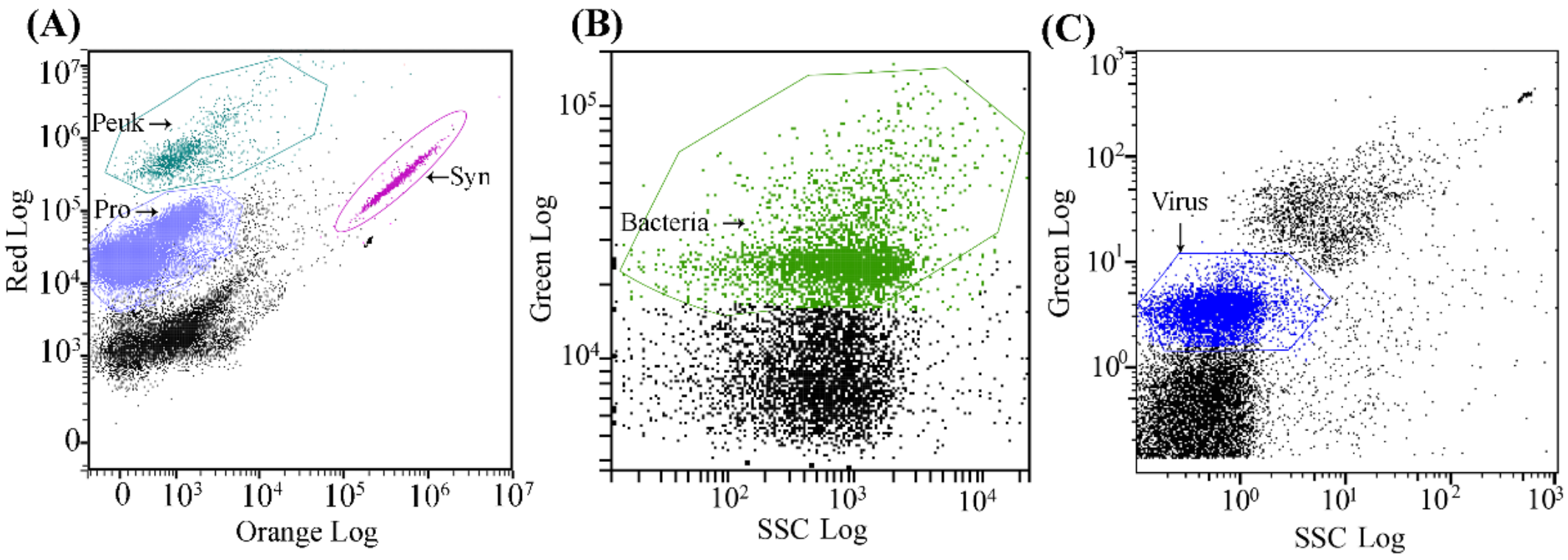

Figure S10. Flow-cytometric identification and gating strategy of microbial groups. Representative cytograms showing side scatter and fluorescence properties of picophytoplankton (A), bacteria (B), and viruses (C). Polygon gates indicate the regions used to discriminate *Prochlorococcus* (Pro), *Synechococcus* (Syn), and picoeukaryotes (Peuk) based on red and orange fluorescence (A), bacteria based on SYBR Green I fluorescence (B), and virus-like particles based on green fluorescence (C). Fluorescent beads were used as internal standards in all analyses.

References

1. Tsai, A. *et al.* Importance of bacterivory by pigmented and heterotrophic nanoflagellates during the warm season in a subtropical western Pacific coastal ecosystem. *Aquat. Microb. Ecol.* **63**, 9–18 (2011).
2. Havskum, H. & Riemann, B. Ecological importance of bacterivorous, pigmented flagellates (mixotrophs) in the Bay of Aarhus, Denmark. *Mar. Ecol. Prog. Ser.* **137**, 251–263 (1996).
3. Unrein, F., Massana, R., Alonso-Sáez, L. & Gasol, J. M. Significant year-round effect of small mixotrophic flagellates on bacterioplankton in an oligotrophic coastal system. *Limnol. Oceanogr.* **52**, 456–469 (2007).
4. Unrein, F., Gasol, J. M., Not, F., Forn, I. & Massana, R. Mixotrophic haptophytes are key bacterial grazers in oligotrophic coastal waters. *ISME J.* **8**, 164–176 (2014).
5. Zhan, Y. *et al.* Spatiotemporal variations of nanophytoplankton mixotrophic strategies in the estuarine-coastal system of the northern South China Sea. *Mar. Pollut. Bull.* **213**, 117622 (2025).
6. Xiao, W. *et al.* Phago-mixotrophic activity within nanophytoplankton community in a subtropical marginal sea. *Limnol. Oceanogr.* **70**, 1636–1650 (2025).
7. Livanou, E. *et al.* Pigmented and heterotrophic nanoflagellates: Abundance and grazing on prokaryotic picoplankton in the ultra-oligotrophic Eastern Mediterranean Sea. *Deep Sea Res. Part II Top. Stud. Oceanogr.* **164**, 100–111 (2019).
8. Anderson, R. & Hansen, P. J. Meteorological conditions induce strong shifts in mixotrophic and heterotrophic flagellate bacterivory over small spatio-temporal scales. *Limnol. Oceanogr.* **65**, 1189–1199 (2020).

9. Li, Q., Dong, K., Wang, Y. & Edwards, K. F. Relative importance of bacterivorous mixotrophs in an estuary-coast environment. *Limnol. Oceanogr. Lett.* **9**, 81–91 (2024).

10. Rocke, E., Pachiadaki, M. G., Cobban, A., Kujawinski, E. B. & Edgcomb, V. P. Protist community grazing on prokaryotic prey in deep ocean water masses. *PLOS One* **10**, e0124505 (2015).

11. Pachiadaki, M. G. *et al.* In situ grazing experiments apply new technology to gain insights into deep-sea microbial food webs. *Deep Sea Res. Part II Top. Stud. Oceanogr.* **129**, 223–231 (2016).

12. Cho, B., Na, S. & Choi, D. Active ingestion of fluorescently labeled bacteria by mesopelagic heterotrophic nanoflagellates in the East Sea, Korea. *Mar. Ecol. Prog. Ser.* **206**, 23–32 (2000).
