# Supplementary material for: Warm-core eddy intensifies surface mixotrophic bacterivory and fuels mesopelagic heterotrophic grazing
Source: Commun Biol. 2026 Jun 8;9:1201. doi: 10.1038/s42003-026-10438-y (PMC13578227; doi:10.1038/s42003-026-10438-y)
Supplement: Supplementary file 2 [file 42003_2026_10438_MOESM2_ESM.docx]

Description of Additional Supplementary Files

File name: Supplementary Data 1

Description: Sampling information for stations in the South China Sea. Each station was sampled at four depths: surface (S), deep chlorophyll maximum (DCM), 200 m, and 500 m.

File name: Supplementary Data 2

Description: Grazing by active phago-mixotrophic nanophytoplankton (PMNP) and heterotrophic nanoflagellates (HNF) in the euphotic and mesopelagic zones reported in various studies.
